# Combining “real effort” with induced effort costs: the ball-catching task

**DOI:** 10.1007/s10683-015-9465-9

**Published:** 2015-09-09

**Authors:** Simon Gächter, Lingbo Huang, Martin Sefton

**Affiliations:** 1 CeDEx and School of Economics, University of Nottingham, Sir Clive Granger Building, University Park, Nottingham, NG7 2RD UK; 2 CESifo, Munich, Germany; 3 IZA, Bonn, Germany

**Keywords:** Real effort task, Piece-rate theory, Team incentives, Gift exchange, Tournaments, Online real effort experiments, C91, C92, J41

## Abstract

We introduce the “ball-catching task”, a novel computerized task, which combines a tangible action (“catching balls”) with induced material cost of effort. The central feature of the ball-catching task is that it allows researchers to manipulate the cost of effort function as well as the production function, which permits quantitative predictions on effort provision. In an experiment with piece-rate incentives we find that the comparative static and the point predictions on effort provision are remarkably accurate. We also present experimental findings from three classic experiments, namely, team production, gift exchange and tournament, using the task. All of the results are closely in line with the stylized facts from experiments using purely induced values. We conclude that the ball-catching task combines the advantages of real effort tasks with the use of induced values, which is useful for theory-testing purposes as well as for applications.

**Electronic supplementary material:**

The online version of this article (doi:10.1007/s10683-015-9465-9) contains supplementary material, which is available to authorized users.

## Introduction

Experiments using real effort tasks enjoy increasing popularity among experimental economists. Some frequently used tasks include, for instance, number-addition tasks (e.g., Niederle and Vesterlund (2007)), counting-zero tasks (e.g., Abeler et al. (2011)) and slider-positioning tasks (Gill and Prowse 2012).^1^ In this paper, we present a novel computerized task, called the “ball-catching task”, which combines a tangible activity in the lab with induced material cost of effort.^2^ In the task, a subject has a fixed amount of time to catch balls that fall randomly from the top of the screen by using mouse clicks to move a tray at the bottom of the screen. Control over the cost of effort is achieved by attaching material costs to mouse clicks that move the tray.

The ball-catching task shares an advantage of real effort tasks in that subjects are required to do something tangible in order to achieve a level of performance, as opposed to simply choosing a number (as is done in experiments that implement cost of effort functions using a pure induced value method, where different number choices are directly linked with different financial costs). A drawback, however, of existing real effort tasks is that in using them the researcher sacrifices considerable control over the cost of effort function. As noted by Falk and Fehr (2003): “while ‘real effort’ surely adds realism to the experiment, one should also note that it is realized at the cost of losing control. Since the experimenter does not know the workers’ effort cost, it is not possible to derive precise quantitative predictions” (p. 404). Incorporating material effort costs re-establishes a degree of control over effort costs and, as we shall demonstrate, allows researchers to manipulate observable effort costs and to make point predictions on effort provision.

Here, we report three studies aimed to evaluate the ball-catching task. In Study 1, we examine individual performance on the ball-catching task under piece-rate incentives. Subjects incur a cost for each mouse click and receive a prize for each ball caught. We first show that clicking behavior corresponds closely to comparative static predictions derived from piece-rate incentive theory. We then estimate the relationship between clicks and catches and use this to predict how the number of clicks will vary as the costs of clicking and the benefits of catching are manipulated. We find that the number of mouse clicks is close to the predicted number of clicks. These findings also add to the literature on empirical testing of incentive theories (Prendergast 1999) by presenting experimental evidence on a tangible task supporting basic piece-rate incentive theory. By comparison, the prominent field evidence reported by Lazear (2000) and lab evidence provided by Dickinson (1999) support comparative static predictions of basic incentive theory, whereas we show that in the ball-catching task the theory also predicts activity levels (number of clicks) accurately.

In Study 2, we demonstrate how the task can be implemented in some classic experiments. We administer the task in experiments used to study cooperation, fairness and competition, namely, team production (e.g., Nalbantian and Schotter (1997)), gift exchange (e.g., Fehr et al. (1993)) and a tournament (e.g., Bull et al. (1987)). In all three experiments, the results reproduce the stylized findings from previous experiments that used purely induced values. Moreover, behavior also follows equilibrium point predictions closely in those experiments where point predictions are available.

In Study 3, we introduce an online version of the ball-catching task and conduct the same experiment as in Study 1 using Amazon Mechanical Turk workers as participants. Comparative statics results are replicated, which we view as an important robustness check. Behavior is noisier than in the lab, however, which most likely is due to the more varied decision environment online compared to the lab.

The remainder of the paper is organized as follows. In Sect. 2 we describe the ball-catching task. In Sects. 3–5 we report the three studies using the task. Section 6 provides a comprehensive discussion of the results of our three studies. Section 7 concludes.

## The ball-catching task

**Fig. 1:**
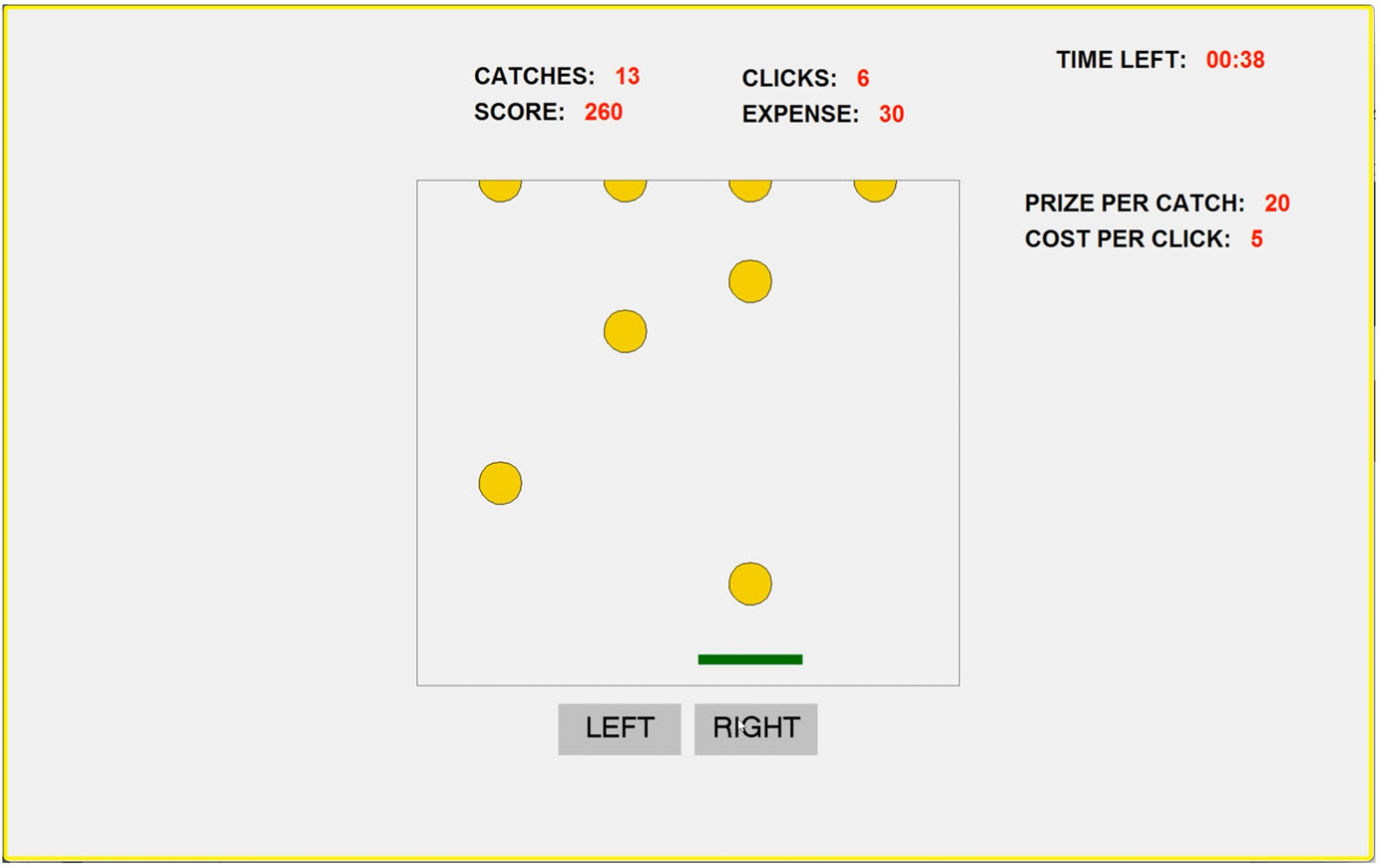
A screenshot of the ball-catching task

The lab version of the ball-catching task is a computerized task programmed in z-Tree (Fischbacher 2007), and requires subjects to catch falling balls by moving a tray on their computer screens. Figure 1 shows a screenshot of the task. In the middle of the screen there is a rectangular task box with four hanging balls at the top and one tray at the bottom. Once a subject presses the “Start the task” button at the lower right corner of the screen, the balls will fall from the top of the task box. In the version used in this paper, the timer starts and balls fall one after another in a fixed time interval. Balls fall at random in each column. The software allows adjusting the speed of falling balls and the time interval between falling balls. It is also possible to change the number of ‘columns’ (i.e., the number of hanging balls) and fix a falling pattern rather than a random one. As will be discussed later, flexibility in all these parameters will allow tight control over the production function in this task, that is, the relationship between the number of balls caught and the number of clicks made.

To catch the falling balls, the subject can move the tray by mouse clicking the “LEFT” or “RIGHT” buttons below the task box. At the top of the screen, the number of balls caught (CATCHES) and the number of clicks made (CLICKS) are updated in real time. We will take the number of clicks as our observable measure of “effort”. As will become clear later, we acknowledge that other forms of effort (e.g., concentration, deliberation) may be exerted by the subject in this task.

Our subjects work on a task that, like all real effort tasks, involves a tangible activity. However, two features distinguish our implementation of the ball-catching task from most real effort tasks: (i) it is approximately costless in terms of physical and cognitive costs required by the task, whereas most real effort tasks involve unobservable physical or cognitive costs; (ii) costs are induced by attaching pecuniary costs to mouse clicks, which implies that, unlike in most real effort tasks, costs are under the control of the experimenter.^3^ By specifying the relation between clicks and pecuniary costs we can implement any material cost of effort function. The most convenient specification might be to use a linear cost function by simply attaching a constant cost to every mouse click, but it is also possible to specify nonlinear cost functions (we will present an example in Sect. 4.2). In the example of Fig. 1 the subjects incurs a cost of 5 tokens for each mouse click. Accumulated costs (EXPENSE) are updated and displayed in real time. It is also possible to attach pecuniary benefits to catches. In Fig. 1 the subject receives 20 tokens for each ball caught and accumulated benefits (SCORE) are updated on screen in real time.

In existing real effort tasks output and effort are typically indistinguishable. In the ball-catching task there is clear distinction between the catches and the clicks variables, with the natural interpretation being that the former represents output and the latter input. Moreover, by choosing the time constraint, ball speed, etc., the researcher has flexibility in selecting the production technology.

Evidence collected in a post-experimental questionnaire suggests that subjects find the ball-catching task easy to understand and learn. In the next section we examine in more detail how subjects perform on the task under piece-rate incentives. In Sect. 5 we present a version of the ball-catching task that can be used for online experiments.

## Study 1: testing the ball-catching task under piece-rate incentives

### Experimental design and comparative static predictions

Study 1 examined performance on the ball-catching task under piece-rate incentives. Each subject worked on the same ball-catching task for 36 periods. Each period lasted 60 s.^4^ In each period one combination of prize-per-catch (either 10 or 20 tokens) and cost-per-click (0, 5 or 10 tokens) was used, giving six treatments that are varied within subjects (see Table 1). We chose a within-subject design to be able to observe reactions to changes in incentives at an individual level. The first 6 periods, one period of each treatment in random order, served as practice periods for participants to familiarize themselves with the task. Token earnings from these periods were not converted to cash. The following 30 periods, five periods of each treatment in completely randomized order (i.e., unblocked and randomized), were paid out for real. In all, 64 subjects participated in the experiment with average earnings of £13.80 for a session lasting about 1 h.^5^

**Table 1:** Within-subject treatments in study 1

Treatment no.	1	2	3	4	5	6
Prize per catch (P)	10	10	10	20	20	20
Cost per click (C)	0	5	10	0	5	10

Given a particular piece-rate incentive, how often would subjects click? Basic piece-rate theory assumes that subjects trade off the costs and benefits of clicking in order to maximize expected utility. Assume that utility is increasing in the financial rewards, which are given by $PQ - Ce$, where $Q$ is the number of catches and $e$ is the number of clicks, and assume the relationship between $Q$ and $e$ is given by $q = f\left( {e,\;\varepsilon } \right)$, where $f$ is a production function with $f^{'} > 0$ and $f^{''} < 0$, and $\varepsilon$ is a random shock uncorrelated with the number of clicks. Given these assumptions the expected utility maximizing number of clicks satisfies:

1 $$ e^{*} = f'\left( {\frac{C}{P}} \right). $$

This analysis posits a stochastic production function linking individual catches and clicks, and so an individual’s optimal number of clicks may vary from trial to trial as the marginal product of a click varies from trial to trial. This may reflect variability in the exact pattern of falling balls from trial to trial. We also recognize that the marginal product function might vary systematically across individuals. To make predictions at the aggregate level, we will estimate the production function (in Sect. 3.3) allowing for individual specific random effects and then use this estimate, evaluated at the mean of the random effects, along with our incentive parameters to predict the average optimal number of clicks. Before we proceed to this estimation, we discuss some features of the optimal number of clicks and how they relate to our experimental design.

The first feature to note is that the optimal number of clicks is homogeneous of degree zero in $C$ and $P$. That is, a proportionate change in both input and output prices leaves the optimal number of clicks unchanged. This feature reflects the assumption that there are no other unobserved inputs or outputs associated with working on the task that generate cognitive or psychological costs or benefits. In fact we can think of two plausible types of unobservable inputs/outputs. First, output may be a function of cognitive effort as well as the number of clicks. For example, output may depend not just on how many clicks a subject makes, but also on how intelligently a subject uses her clicks. If the production function is given by $f\left( {e,\;\kappa ,\;\varepsilon } \right)$, where $\kappa$ represents cognitive effort, then $e^{*}$ will reflect a trade-off between $e$ and $\kappa$. If *all* input and output prices were varied in proportion (including the “price” of $\kappa$), the optimal number of clicks would be unaffected. However, a proportionate change in just *C* and *P* would affect $e^{*}$. If $e$ and $\kappa$ are substitute inputs then a proportionate increase in $C$ and $P$ will result in a decrease in $e^{*}$ as the subject substitutes more expensive clicking with more careful thinking. Second, subjects may enjoy additional psychological benefits from catching balls. For example, suppose that in addition to the pecuniary costs and benefits there is a non-monetary benefit from a catch, and suppose this psychological benefit is worth *B* money-units per catch. Again, proportionate changes in *P*, *C* and *B* would leave optimal effort unchanged, but a change in just *P* and *C* would not. Maximization of $(P + B)Q - Ce$ implies that a proportionate increase in $C$ and $P$ (holding *B* constant) will decrease $e^{*}$.

Our experimental treatments allow us to test whether unobservable costs/benefits matter compared with induced effort costs in the ball-catching task. Our design includes two treatments that vary $C$ and $P$ while keeping the ratio $\frac{C}{P}$ constant (treatments 2 and 6 in Table 1). In the absence of unobserved costs/benefits, the distribution of clicks should be the same in these two treatments. The presence of unobserved costs/benefits could instead lead to systematic differences. Note that with this design the prediction that optimal clicking is homogeneous of degree zero in *C* and *P* can be tested without the need to derive the underlying production function, $f$, since all that is needed is a comparison of the distributions of clicks between these two treatments.

A second feature of the optimal number of clicks is that, for positive costs of clicking, the optimal number of clicks decreases with the cost-prize ratio. Our design includes four further treatments that vary this ratio. Comparisons between treatments with different cost-prize ratios allow simple tests of the comparative static predictions of piece-rate theory, again without the need to estimate the production function. The variation in incentives across treatments serves an additional purpose: it allows us to recover a more accurate estimate of the underlying production function over a wide range of clicks.

A final feature of the optimal solution worth noting is that when the cost-per-click is zero optimal clicking is independent of *P*. In this case, since clicking is costless the individual’s payoff increases in the number of catches, and so regardless of the prize level the individual should simply catch as many balls as possible. Again, if there are psychological costs/benefits associated with the task this feature will not hold. Indeed, one could use the ball-catching task without material costs of clicking, basing comparative static predictions (e.g. that the number of catches will increase as the prize per catch increases) on psychological costs of effort. However, like in many existing real effort tasks, the ball-catching task without material costs might exhibit a “ceiling effect”, that is unresponsiveness of the number of clicks to varying prize incentives.^6^ For this reason our design includes two treatments where the material cost of clicking is zero (treatments 1 and 4 in Table 1). These allow us to test whether there is a ceiling effect in the ball-catching task without induced clicking costs.

### Comparative statics results

**Fig. 2:**
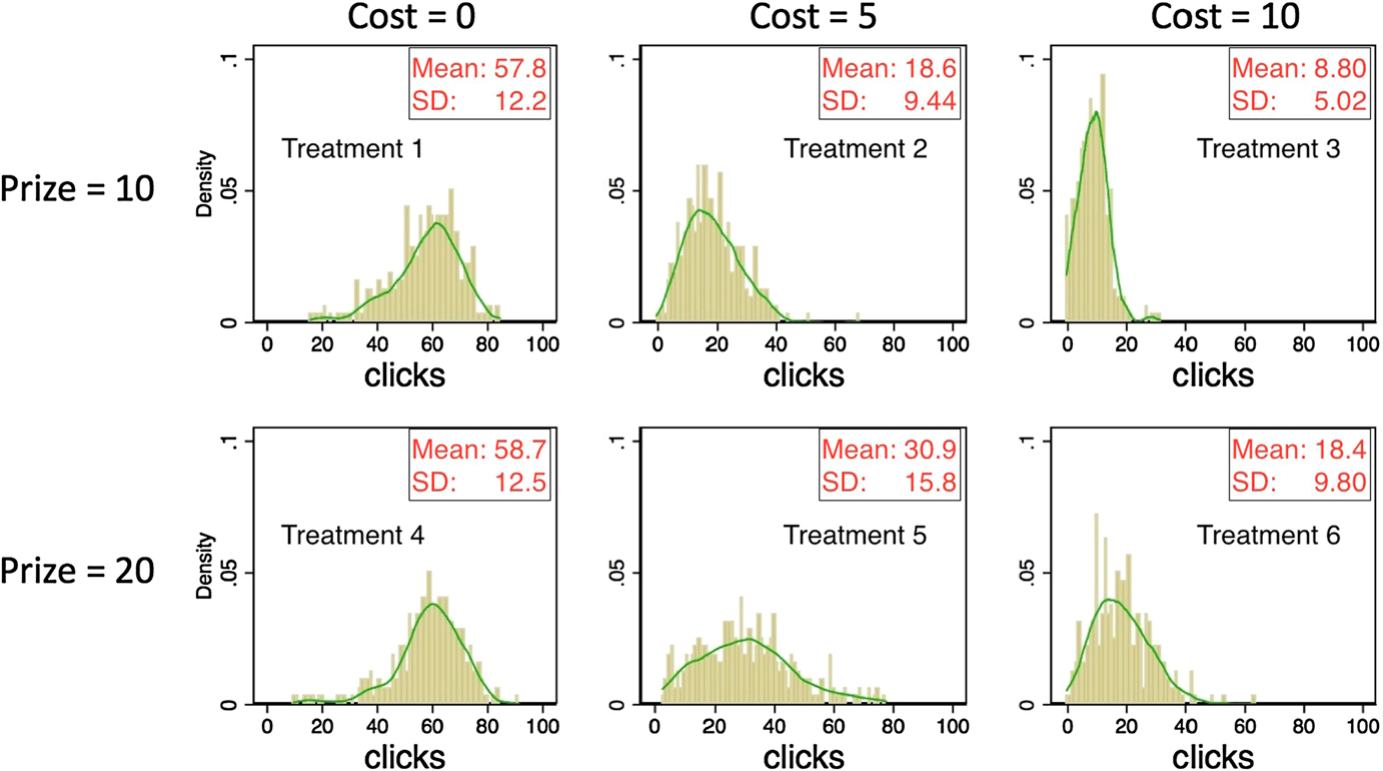
Distributions and kernel density distributions of the number of clicks in Study 1

Figure 2 shows the distributions of clicks for each treatment, pooling over all subjects and periods. Clear differences between panels show that clicking behavior varies across incentive treatments. We begin by examining how these differences relate to the comparative static predictions based on optimal clicking (1).^7^

Consider first the comparison between treatments 2 (P = 10, C = 5) and 6 (P = 20, C = 10). These treatments vary the financial stakes without altering the cost/prize ratio. The basic piece-rate theory prediction is that this will not have a systematic effect on clicking. As discussed in Sect. 3.1 however, unobserved psychological costs/benefits associated with the task will lead to systematic differences between the distributions of clicks in the two treatments. We find that the distributions of clicks are very similar, with average clicks of 18.6 under low-stakes and 18.4 under high stakes. Using a subject’s average clicks per treatment as the unit of observation, a Wilcoxon matched-pairs signed-ranks test (p = 0.880) finds no significant difference between treatments 2 and 6. Thus, we cannot reject the hypothesis that the average number of clicks is invariant to scaling up the financial stakes.

Next we ask whether variation in the cost-prize ratio affects clicking as predicted. Will increasing the cost-per-click, holding the prize-per-catch constant, reduce the number of clicks? And will the number of clicks depend on the prize level for a given clicking cost? First, we compare the top three panels of Fig. 2, where the prize is always 10. We observe a clear shift of the distribution of the number of clicks when moving across treatments with lowest to highest induced clicking costs. The average number of clicks falls from 58.7 to 18.6 to 8.8 as the cost-per-click increases from 0 to 5 to 10. Friedman tests for detecting systematic differences in matched subjects’ observations, using a subject’s average clicks per treatment as the unit of observations, show that the differences across treatments are highly significant (p < 0.001). A similar pattern is observed in the bottom three panels, where the prize is always 20, and again the differences are highly significant (p < 0.001).

Next, we perform two vertical comparisons between treatments 2 and 5 and between treatments 3 and 6. Holding the clicking costs constant, we find that a higher prize leads to higher number of clicks in both comparisons (Wilcoxon matched-pairs signed-ranks test: p < 0.001).

Finally, a comparison between treatments 1 and 4 offers an examination of whether a ceiling effect, observed in many real effort tasks, is present in the ball-catching task. In these treatments the cost-per-click is zero, but the prize-per-catch is 10 in treatment 1 and 20 in treatment 4. If there is no “real” psychological cost/benefit associated with working on the task, subjects should simply catch as many balls as possible and we should observe the same distribution of the number of clicks in these two treatments, thus exemplifying the typical ceiling effect. Comparing the distributions of clicks across the zero-cost treatments illustrated in Fig. 2 suggest that distributions are very similar. Average clicks are 57.8 in the low prize treatment and 58.7 in the high prize treatment. The closeness of average clicking between treatments 1 and 4 is statistically supported by a Wilcoxon matched-pairs signed-ranks test (p = 0.215), again using a subject’s average clicks per treatment as the unit of observation. The sharp contrast between the strong prize effect in treatments with induced effort costs and the absence of a prize effect in the zero-cost treatments illustrates that the ceiling effect can be avoided by incorporating financial costs in the ball-catching task.^8^

In sum, as stated in the following result, we find that the comparative static predictions of basic piece-rate theory are borne out in the experimental data.

***Result 1:The main comparative statics predictions are supported:***
1. *Varying the financial stakes without altering the cost//prize ratio does not affect clicking behavior.*
2. *Increasing the cost-per-click while keeping the prize-per-catch constant reduces the number of clicks; increasing the prize-per-catch while keeping the cost-per-click constant increases the number of clicks.*
3. *When the cost-per-click is zero, the value of the prize-per-catch does not affect clicking behavior (ceiling effects).*

Our next goal is to derive point predictions about the number of clicks in the various treatments and to compare them to the data. To be able to do so, we next estimate the production function, which we will then use to derive the point predictions.

### The production function

Our empirical strategy for estimating the production function is to first specify a functional form by fitting a flexible functional form to the catches-clicks data using the full sample. Next, we estimate the production function, allowing for persistent as well as transitory unobserved individual effects and fixed period effects. We then test whether the production function is stable across periods and invariant to varying prize levels. We will also examine the stability of the production function across experimental sessions.

**Fig. 3:**
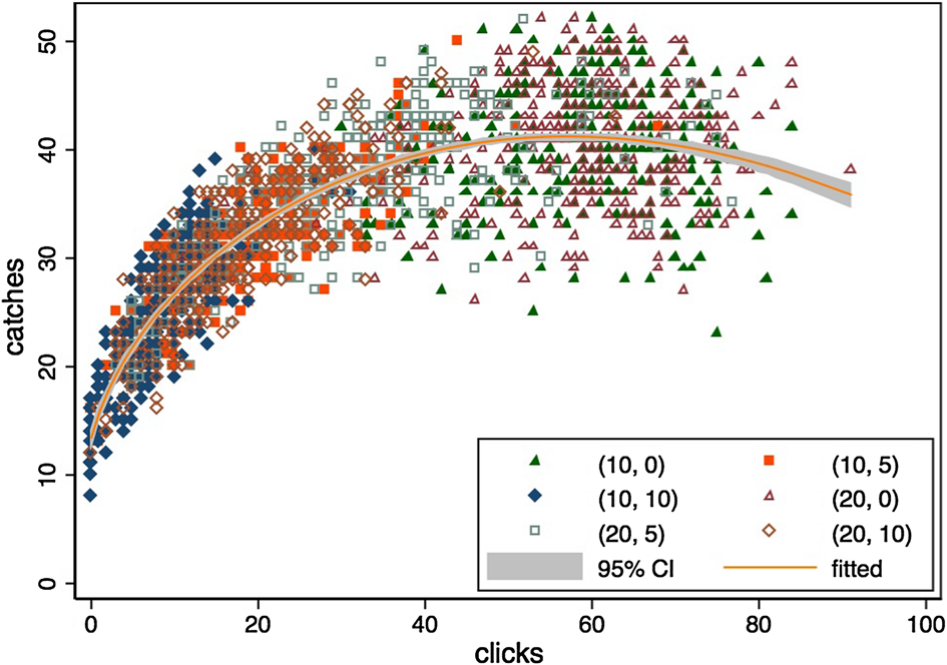
The relation between clicks and catches and the estimated production function. *Note* the first entry in (*, *) denotes the prize per catch and the second the cost per click. The fitted production functional form is given by $Q = 9.507 + 5.568e^{0.5} - 0.003e^{2}$, where $Q$ denotes the number of catches and $e$ the number of clicks. The estimates of coefficients are from a fractional polynomial regression

Figure 3 shows the observed catches-clicks data from all treatments along with a fitted production function based on a fractional polynomial regression.^9^ The fitted production function has a clear concave shape, indicating a diminishing marginal rate of return to clicks. After a point, the production function is decreasing, indicating that there is a “technological ceiling” beyond which more clicking may actually lead to lower production levels. Observations in the decreasing range are predominantly from the treatments with a zero cost of clicking. As one of the main advantages of using the ball-catching task is precisely that clicking can be made costly, the decreasing part of the production function should be of little concern, since with positive clicking costs the number of clicks will be within the range where the empirical production function is concave and increasing.

Using the functional form suggested by the fractional polynomial regression, we move on to estimate the following random coefficients panel data model:

$$ Catches_{i,r} = \beta_{0} + \beta_{1} Clicks_{i,r}^{0.5} + \beta_{2} Clicks_{i,r}^{2} + \left( {\delta_{r} + \omega_{i} + u_{i,r} } \right)Clicks_{i,r}^{0.5} ,             (2) $$

where $Catches_{i,r}$ and $Clicks_{i,r}$ are respectively the number of catches and the number of clicks of subject $i$ in period $r$. Period dummies $\delta_{r}$ (with the first period providing the omitted category), an individual random effect $\omega_{i}$ with mean zero and variance $\sigma_{\omega }^{2}$, and a random error $u_{i,r}$ with mean zero and variance $\sigma_{u}^{2}$ are all assumed to be multiplicative with $Clicks_{i,r}^{0.5}$. Our specification of multiplicative heterogeneity and heteroskedasticity allows both persistent and transitory individual differences in the *marginal product function,* which could also vary across periods. The model thus predicts heterogeneity in clicking both across and within subjects.^10^ All equations are estimated using maximum likelihood and estimates are reported in Table 2.^11^

**Table 2:** Panel data regressions for model (2) in study 1

Dep. var. catches	Coefficient estimates (SE)		
	(1) full sample	(2) Prize = 10	(3) Prize = 20
Intercept	10.107*** (0.230)	10.477*** (0.308)	9.405*** (0.423)
Clicks^0.5^	5.495*** (0.132)	5.402*** (0.216)	5.660*** (0.171)
Clicks^2^	−0.003*** (0.000)	−0.003*** (0.001)	−0.003*** (0.000)
$\sigma_{\omega }$	0.366*** (0.038)	0.352*** (0.045)	0.384*** (0.042)
*σ*_*u*_	0.796*** (0.013)	0.870*** (0.021)	0.694*** (0.016)
N	1905	946	959

All period dummies are included and insignificant except for period 2 using the full sample. *** p < 0.01

Columns (1), (2) and (3) in Table 2 reports the coefficient estimates for the full sample, the sub-sample with the prize of 10 and the sub-sample with the prize of 20 respectively. Note the similarity between the estimates of the parameters of the production function in all equations. The fitted production functions for the two sub-samples with different prizes are shown in Fig. B1 in the online supplementary materials: the two production functions almost coincide. Furthermore, we find that both persistent and transitory unobserved individual effects are statistically significant, and that the transitory unobservables account for more of the variation in clicking than the persistent individual differences.

To formally test whether the production function is invariant to different prize levels, we proceed to estimate an augmented model by adding interactions of the intercept, covariates $Clicks^{0.5}$ and $Clicks^{2}$ with a binary variable indicating whether the prize is 10–20. We then perform a likelihood ratio test of the null hypothesis that the coefficients on the interaction terms are all zero. We cannot reject the null hypothesis, indicating that the production function is stable across prize levels ($\chi^{2} \left( 3 \right)$ = 4.70, p = 0.195).

To test the stability of the production function across experimental sessions, we estimate an augmented model by adding interactions of the intercept, $Clicks^{0.5}$ and $Clicks^{2}$ with a session dummy. We cannot reject the null hypothesis that the production function is invariant across sessions ($\chi^{2} \left( 3 \right)$ = 2.60, p = 0.458). In fact the fitted production functions are barely distinguishable.^12^ We summarize these findings in the following result.

*Result 2:* *The estimated production function, that is, the relationship between catches and clicks, is increasing in clicks and concave. The production function is stable across different prize levels as well as across different experimental sessions.*

### Comparing the predicted and actual number of clicks

With the estimated production function from model (2) and treatment parameters, we are ready to see how quantitative predictions on clicking perform.

Table 3 compares the predicted number of clicks that is derived from Eq. (1) given the estimated production function reported in column (1) of Table 2 and the cost-prize parameters, with the actual number of clicks for every treatment.^13^ We find that average actual clicks are very similar to the predicted number of clicks in treatments 1, 2, 4 and 6 and near to, but statistically significantly different from, predicted clicks in treatments 3 and 5 (subjects seem to have over-clicked in treatments 3 and under-clicked in treatment 5).^14^ Thus, overall, not only did they change their clicking behavior in the predicted direction when incentives changed, but also for given incentives their clicking was close, on average, to the profit maximizing level. The results are surprising given that subjects cannot know the production function *a priori* and therefore are in no position to calculate the optimal level of clicking. Nonetheless, on average, they behaved *as if* they knew the underlying structural parameters and responded to them optimally. These findings are summarized in our next result.

**Table 3:** Comparisons between the predicted number of clicks and the actual number of clicks

Treatment no.	1	2	3	4	5	6
Prize per catch (P)	10	10	10	20	20	20
Cost per click (C)	0	5	10	0	5	10
Predicted clicks	57.4	19.5	6.9	57.4	34.5	19.5
Av. actual clicks (SD)	57.8 (12.2)	18.6 (9.44)	8.8 (5.02)	58.7 (12.5)	30.9 (15.8)	18.4 (9.80)
p-value	0.723	0.367	0.000	0.276	0.040	0.294

p values are based on two-tailed one-sample t-tests using a subject’s average clicks per treatment as the unit of observation when testing against the predicted clicks

*Result 3: The average number of clicks is close to the point prediction in all treatments but deviates statistically significantly from point predictions in treatments 3 and 5.*

**Fig. 4:**
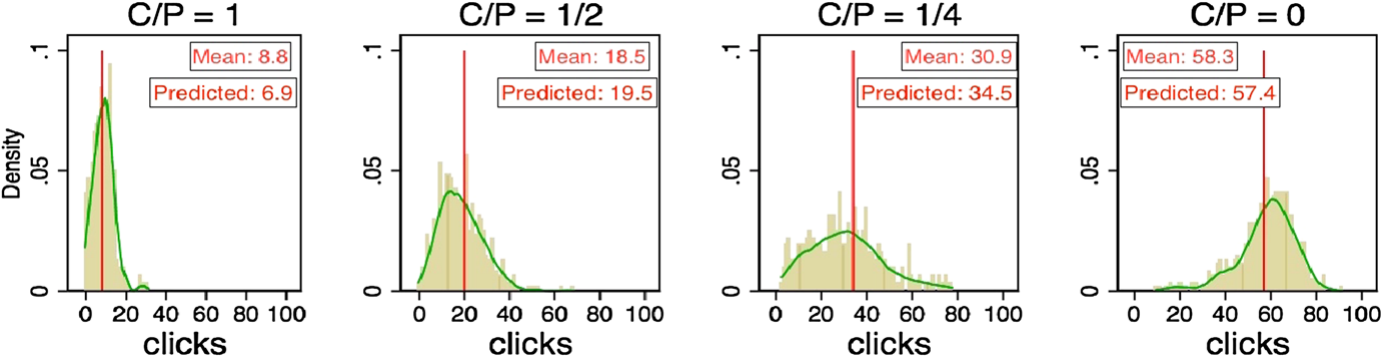
Distributions and kernel density distributions of the actual number of clicks and the predicted clicks. *Note* the *vertical line* in each panel represents the predicted number of clicks

Figure 4 shows the predicted clicks and the distribution of actual clicks by combining categories whenever the treatments have the same predicted clicks. The distribution of clicks is approximately centered on the predicted clicks in each case, but shows variability in clicking for any given *C/P* ratio. As noted earlier, if the marginal product of clicking is subject to individual-specific and idiosyncratic shocks variability in clicking is to be expected.

In the next section, we provide further tests for the suitability of the ball-catching task by investigating its performance in well-known experimental settings that hitherto have typically used induced-value designs. This will be a further opportunity to see whether the ball-catching task produces behavior that is consistent with equilibrium comparative static or point predictions.

## Study 2: applications—team production, gift exchange and tournament

The previous section has demonstrated the accuracy of predictions on clicking using the ball-catching task in an individual decision making task. In this section, we use the ball-catching task in three classic interactive experiments that have been used to study cooperation, reciprocity, and competition. We chose these applications for several reasons. First, they represent important classes of experimental games using induced value designs. Second, they allow for further tests of theoretical point predictions and/or of comparative static predictions in interactive settings. Third, they illustrate the versatility of the ball-catching task with regard to manipulations of the production function and the induced values for the cost function. We will utilize the estimated production function from Study 1 to derive predictions on clicking whenever possible.

We ran five sessions, each with 32 subjects, for a total of 160 subjects. In each session two unrelated treatments were conducted, each involving ten repetitions of a task. Details of the treatments are specific to each session and will be explained separately below. Instructions for the second treatment were given after the first treatment was completed. At the end of each session, a post-experimental questionnaire was administered asking for subjects’ perception of the ball-catching task, including its difficulty, enjoyableness and boredom. All the sessions were run at the CeDEx lab at the University of Nottingham. Sessions lasted no more than one hour and the average earnings were around £13.00.^15^

### Team production

The understanding of free-riding incentives in team production is at the heart of contract theory and organizational economics (Holmstrom 1982). A standard experimental framework for studying team production is the voluntary contribution mechanism in which the socially desirable outcome is in conflict with individual free-riding incentives (see a recent survey in Chaudhuri (2011) in the context of public goods).

Our team production experiment was run over three sessions. One session included a team production (TP) treatment, in which four team members worked on the ball-catching task independently over 10 periods. The same four subjects played as a team for the entire 10 periods. For each ball caught, the subject contributed 20 tokens to team production while he/she had to bear the cost of clicking, with a cost per click of 5 tokens. At the end of each period, total team production was equally shared among the four team members. Each member’s earnings were determined by the share of the production net of the individual cost of clicking. Note that an individual’s marginal benefit from another catch is 5 tokens, whereas the marginal benefit accruing to the entire group is 20 tokens. The other two sessions included control treatments where individuals play 10 periods according to a simple individual piece-rate. In the first treatment (PR20) an individual receives a prize per catch of 20 tokens and incurs a cost per click of 5 tokens. The second treatment (PR5) has a prize per catch of 5 tokens and a cost per click of 5 tokens.

The amount of clicking in PR5 gives a “selfish” benchmark for the TP treatment, while clicking behavior in PR20 gives an “efficiency” benchmark. If a subject in the TP treatment is only concerned about her own private costs and benefits from clicking and catching, and equates marginal costs to marginal private benefits, she should click the same as in PR5. On the other hand, if she is concerned about total team production and equates marginal costs to marginal social benefits, then she should provide the same clicks as in PR20. Our hypothesis is that free-riding incentives would drive clicking towards the selfish benchmark, as is observed in many similar experiments using induced values (e.g., Nalbantian and Schotter (1997) and many public goods experiments using voluntary contribution mechanisms).

**Fig. 5:**
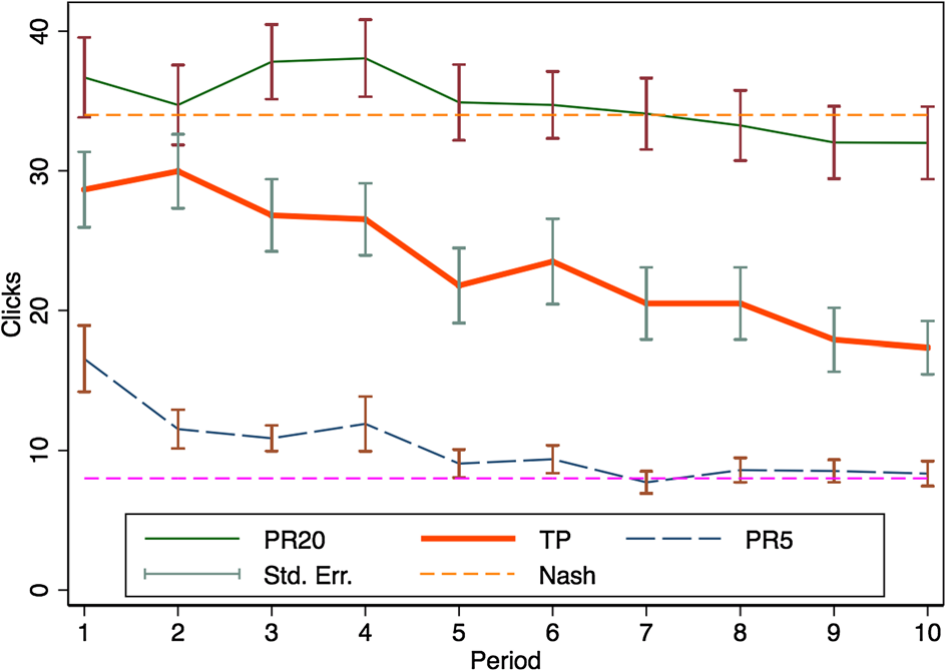
Average clicks over time in team production

Figure 5 displays the average numbers (± 1 SEM) of clicks in the three treatments. The two horizontal lines represent the Nash predictions on optimal clicking levels in PR20 and PR5 respectively (using the estimated production function from Study 1 to compute the optimal clicking levels).

The figure shows a clear declining average number of clicks over time in TP. Average clicks decrease from 30 clicks to just above 17 clicks in the last period. By comparison, average clicks in PR20 decrease from 38 to 32 and in PR5 from 16 to 8 and thus is consistent with our findings in Study 1. Subjects in TP under-provide effort, relative to the efficiency benchmark, from the very first period and steadily decrease their clicking. Even in the final period, however, average clicks exceed the extreme selfishly optimal level. This empirical result is qualitatively similar to previous findings from experiments using induced values, such as Nalbantian and Schotter’s (1997) revenue sharing treatment and many public goods experiments, and also from some real effort experiments on team incentives (e.g., Corgnet et al. (2015)).

### Gift exchange

The gift exchange experiment (Fehr et al. 1993) examines reciprocal behavior between subjects in the role of firms and subjects in the role of workers. The gift exchange game using induced value techniques has been a workhorse model for many experimental investigations of issues in labor economics and beyond (see Gächter and Fehr 2002; Charness and Kuhn 2011 for surveys).

Our version of the bilateral gift exchange experiment follows Gächter and Falk (2002), except that they used induced values whereas we use the ball-catching task and slightly different parameters which we deem more suitable for the present purpose. In our experiment, in each period the firm offers a wage between 0 and 1000 tokens to the matched worker who then works on the ball-catching task. Each ball caught by the matched worker adds 50 tokens to the firm’s payoff. The worker’s payoff is the wage minus the cost of clicking. To compensate for possible losses, every firm and worker received 300 tokens at the beginning of each period. We implemented the gift exchange game in two sessions, one using a treatment with stranger matching over ten periods and the other using a treatment with partner matching over ten periods.

We made two key changes to the task compared with the version used in Study 1. First, we reduced the number of balls that could be caught within 60 s from 52 to 20 by increasing the time interval between falling balls. We made this change because we wanted to reduce the influence of random shocks as much as possible. The change makes it easy for a subject to catch every ball so that reciprocal behavior by workers could be reflected in their clicks as well as in their actual outputs. Second, the cost schedule was changed to a convex function in accordance with the parameters used in most gift exchange experiments. The cost for each click is depicted in Table 4. For example, the 1st and 2nd clicks cost 5 tokens each, the 3rd click cost 6 tokens, etc., and finally the last column with No. 30 + means that the 30th and any further clicks cost 12 tokens each. If, for example, the worker makes a total of three clicks she will incur a total cost of 5 + 5 + 6 = 16 tokens.

**Table 4:** The cost schedule in gift exchange

No. of click	1	2	3	4	5	6	7	8	9	10	11	12	13	14	15
Cost	5	5	6	6	6	7	7	7	7	8	8	8	8	8	9
No. of click	16	17	18	19	20	21	22	23	24	25	26	27	28	29	30+
Cost	9	9	9	9	10	10	10	10	10	11	11	11	11	11	12

**Fig. 6:**
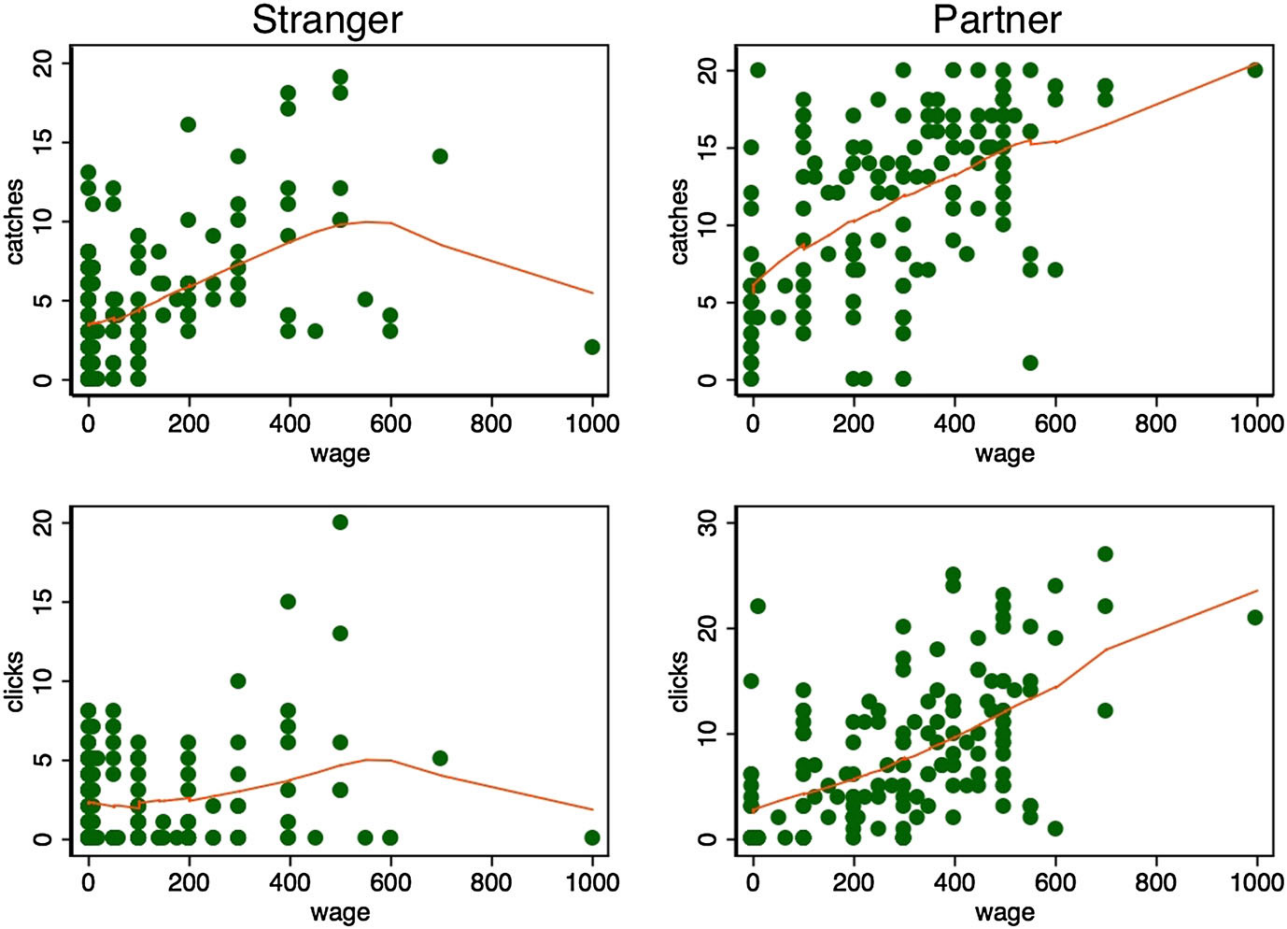
Reciprocal patterns in gift exchange. The *upper* panel shows the relationship between outputs and wages in both treatments and the *lower* panel displays the relationship between clicks and wages. The relationship in the stranger matching treatment is shown in the *left* panels and in the partner matching treatment in the *right* panels. The fitted lines are estimated from non-parametric lowess regressions with the bandwidth equal to 0.8

Based on many gift exchange experiments and in particular the results by Gächter and Falk (2002) and Falk et al. (1999) who also compared partners and strangers in gift exchange, we expect gift exchange and predict that the reciprocal pattern is stronger with partner matching where it is possible to build up a reputation between a firm and a worker. Figure 6 confirms both predictions. It shows the relationship between outputs and wages on the upper panel and the relationship between clicks and wages on the lower panel. The data suggests a clear reciprocal pattern in both treatments and an even stronger pattern in the partner treatment whether we look at outputs or clicks.

For formal statistical tests we estimate the following random effects panel data model for the number of clicks on the wage received:

$$ Click_{i,r} = \beta_{0} + \beta_{1} wage_{i,r} + \omega_{i} + \delta_{r} + u_{i,r} $$

where $\omega_{i}$ is an individual-specific random effect identically and independently distributed over subjects with a variance $\sigma_{\omega }^{2}$, $\delta_{r}$ denotes a period dummy for the $r$ ^th^ period (with the first period providing the omitted category), and $u_{i,r}$ is a disturbance term, assumed to be identically and independently distributed over subjects and periods with a variance $\sigma_{u}^{2}$.

Table 5 reports the estimates for both treatments and also for the pooled sample with an additional interaction term. Consistent with gift exchange reciprocity and the graphical evidence from Fig. 6, workers in both treatments respond to higher wages by clicking more, and the number of clicks differs systematically from zero clicks. Furthermore, the strength of reciprocity is stronger with partners than strangers as the interaction term between the wage received and the treatment dummy in the column (3) is highly significant. These results in our ball-catching gift exchange experiment are qualitatively similar to findings from induced value experiments in Falk et al. (1999) and Gächter and Falk (2002). Our results from the stranger treatment are also consistent with an early real effort gift exchange experiment by Gneezy (2002) who used a maze solving task (without induced values) to measure worker’s performance, although Gneezy’s experiment was conducted in a one-shot setting.

**Table 5:** Random effects regressions for worker’s clicks in gift exchange

Dep. var.: clicks	Coefficient estimates (SD)		
	(1) Stranger	(2) Partner	(3) Pooled
Wage	0.003** (0.001)	0.014*** (0.003)	0.004** (0.002)
Partner			1.154 (0.797)
Wage × partner			0.014*** (0.003)
Intercept	3.279*** (0.681)	3.746*** (1.444)	2.200** (0.952)
$\sigma_{\omega }$	1.753	3.346	2.293
*σ*_*u*_	2.649	4.397	3.972
Hausman test for random versus fixed effects	df = 10 p = 1.000	df = 10 p = 0.956	df = 11 p = 0.984
N	160	160	320

All period dummies are statistically insignificant. Partner is a dummy which equals 1 if the treatment is the partner matching and 0 if the stranger matching. *** p < 0.01, ** p < 0.05

### Tournament

Tournament incentive schemes, such as sales competitions and job promotions, are an important example of relative performance incentives (Lazear and Rosen 1981). One early laboratory experiment by Bull et al. (1987) found that tournament incentives indeed induced average efforts in line with theoretical predictions. But the variance of behavior was much larger under tournament incentives than under piece-rate incentives. Many induced value tournament experiments have been conducted since (see Dechenaux et al. (2014) for a survey).

In one session we included a simultaneous tournament treatment. The 32 subjects were randomly matched into pairs in a period and each pair competed in the ball-catching task for a prize worth 1200 tokens. The winner earned 1200 tokens net of any cost of clicking, whereas the loser received 200 tokens net of any cost of clicking. The cost per click was always 5 tokens. Each player’s probability of winning followed a piecewise linear success function (Che and Gale 2000; Gill and Prowse 2012): *prob*{*win*} = (own output – opponent’s output + 50)/100. This procedure was repeated over 10 periods.

We use this contest success function because it allows us to make a point prediction on the expected number of clicks. This is because the specified piecewise linear success function implies that an additional catch increases the probability of winning by 1/100. Thus, the marginal benefit of clicking is equal to the prize spread between the winner prize and the loser prize, 1000, multiplied by 1/100, multiplied by the marginal product of a click. The marginal cost of clicks is 5 tokens. Once again, we utilize the estimated production function from Study 1 to compute the optimal number of clicks, which turns out to be 20 clicks. Notice that while an additional catch increases earnings by 10 tokens in treatment 2 of Study 1, here an additional catch increases *expected* earnings by 10 tokens.

**Fig. 7:**
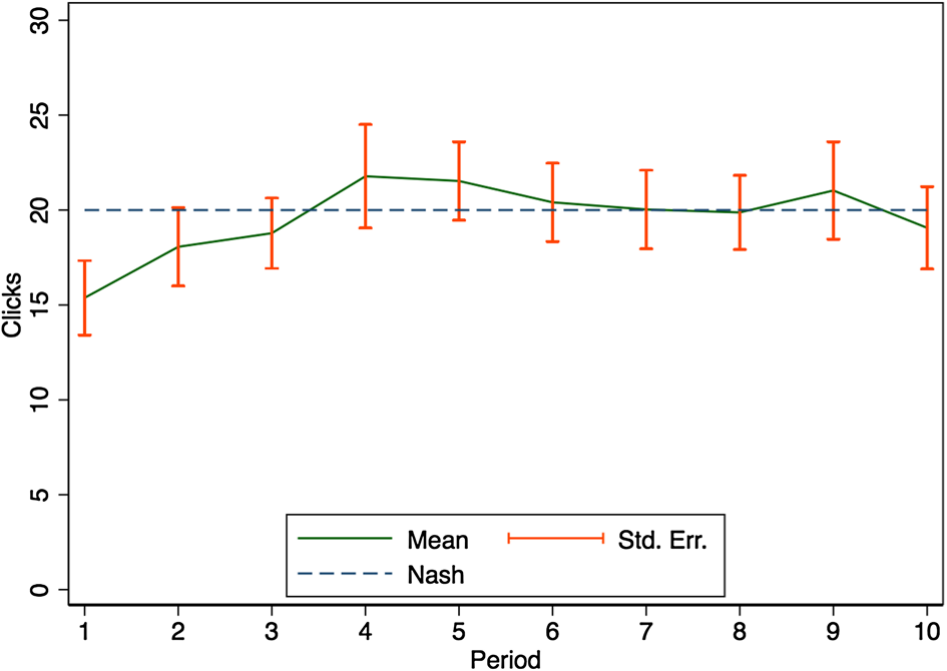
Average clicks over time in tournament

Figure 7 displays the average clicks (± 1 SEM) across all subjects and periods. We observe quick convergence to the predicted clicking level. The variance of clicks in tournament also appears to be larger than that observed in treatment 2 of Study 1. The standard deviation of clicks is around 12 in the former and 9.4 in the latter, perhaps reflecting the stochastic nature of the relationship between catches and earnings under tournament incentives.^16^ Both results are qualitatively similar to previous findings from Bull, et al. (1987).

## Study 3: an online version of the ball-catching task

### The ball-catching task on Amazon Mechanical Turk

As a third test of the versatility of the ball-catching task, we introduce an online version. This online version is programmed in PHP and has been designed to resemble the lab version as closely as possible.^17^ The purpose of this section is to show the potential (and limitations) of using the ball-catching task in online experiments, which increasingly appear to be a valuable complement to experiments in the physical laboratory.

We ran the same experiment as in Study 1 on Amazon Mechanical Turk (MTurk; see the supplementary materials for instructions).^18^ In total, we recruited 95 subjects from MTurk and 74 of them finished the task. Recruitment took around 10 min. Given the unusually long duration of the task (50 min), the 78% completion rate suggests that our promised payment is sufficiently attractive to most of the workers on MTurk. The average payment, including a $3 participation fee, was around $5.90, which was well above what most MTurk tasks offered. The average age was 35 years, ranging from 20 to 66 years; and 52% were male.

**Fig. 8:**
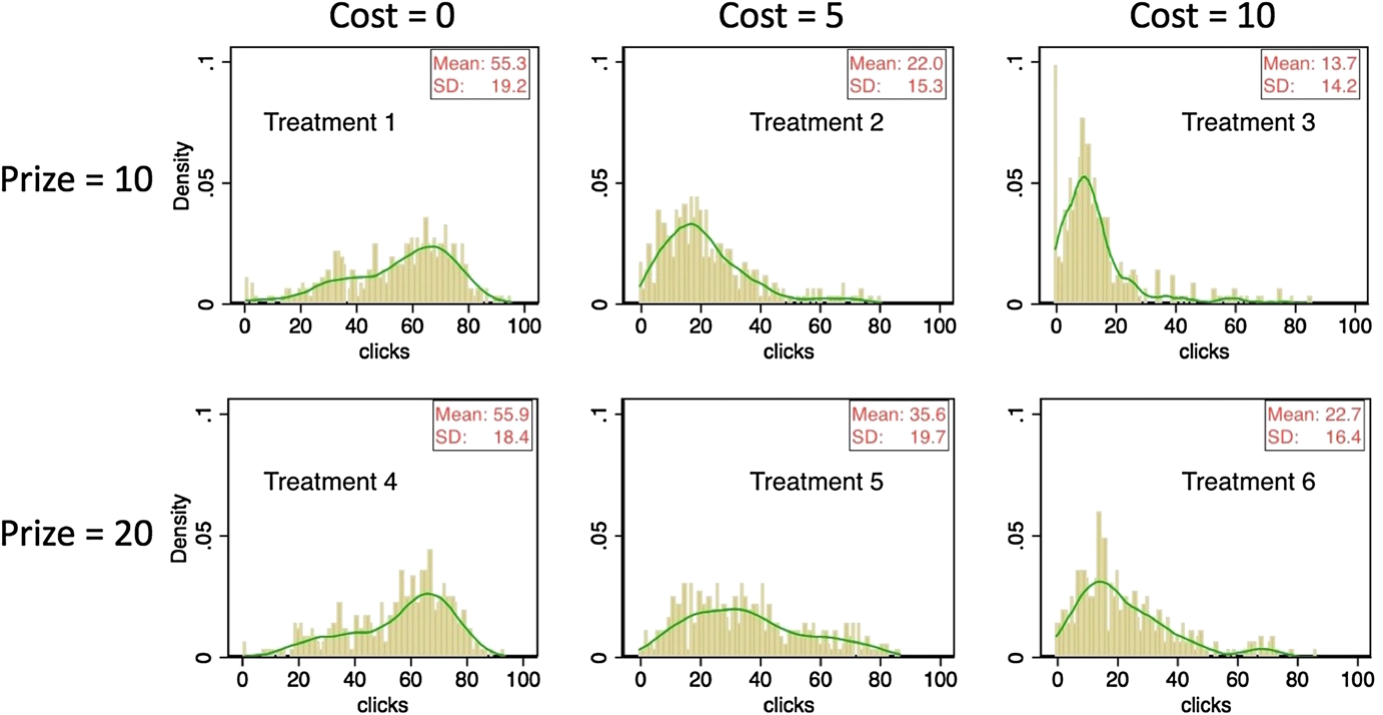
Distributions and kernel density distributions of the number of clicks in study 3

Paralleling the presentation of Study 1 results, Fig. 8 summarizes the distribution and the kernel densities of the number of clicks for each treatment. In general, we find that the comparative statics results are very similar to those in Study 1. A Wilcoxon signed-ranks test using a subject’s average clicks per treatment as the unit of observations suggests that homogeneity of degree zero also holds here: the difference in clicks between the two treatments with the same *C/P* ratio is not systematic (p = 0.309). The same is true for the difference in clicks between the two treatments with *C* = 0 (p = 0.832). Similarly, when comparing treatments with the same prize, Friedman tests indicate that comparative static predictions for different costs are supported (p < 0.001 in both comparisons).

We observe some notable differences between the online and the lab version. The variance of clicking in each treatment for MTurkers appears to be higher than in the lab with student subjects. Moreover, we find that the production function is not invariant to prize levels, nor is it stable across sub-samples, thus preventing us from making meaningful point predictions.^19^

## Discussion

Real effort tasks have the advantage that they offer subjects something tangible to do rather than just choosing among abstract options. The potential cost to the experimenter is loss of control because subjects might experience unobserved psychological benefits or costs. Thus, there is a tradeoff between “realism” and experimental control. The ball-catching task mitigates this tradeoff because it allows for a tangible activity *and* control over important parameters, such as the production function and the cost function. This feature is particularly important if the experimenter wants to test theoretical predictions, in particular, point predictions. Existing real effort tasks typically allow at best for comparative static predictions, but not point predictions, because the latter requires full control over *all* costs and benefits, be they material or psychological.

Psychological costs and benefits always exist to some degree because *any* decision environment inevitably triggers emotions and requires some cognitive effort. Arguably, these psychological effects are stronger in real effort experiments than in abstract induced value settings. Smith (1982) (in particular pp. 930–934) was well aware of these non-monetary costs and benefits and argued that the “precepts” of induced value experiments will provide the necessary control of the experimental environment. The precepts are *non*-*satiation* in the reward medium (money), *salience* (rewards in the experiments should depend on decisions), and in particular *dominance* (the “reward structure dominates any subjective costs (or values) associated with participation in the activities of the experiment” (p. 934)). It is the control over costs and benefits that renders experiments an informative tool to test economic theories – be it an abstract induced value experiment or a real effort experiment. Satisfying *dominance* may be harder to achieve in real effort experiments than in induced value experiments.

Thus, the usefulness of the ball-catching task to test economic theories requires that dominance holds: psychological costs and benefits should be relatively small and dominated by pecuniary payoff considerations. In our piece-rate setting, “small” means that, in a statistical sense, clicks should be homogeneous of degree zero in those costs and prizes, which the experimenter can manipulate. Our results in Study 1 unambiguously support this requirement. Thus, the ball-catching task has passed a first important test for its usefulness to test economic theories.

As a second test, we derived further comparative static predictions about how clicking levels should vary with changing costs and prizes. The results strongly support the comparative static predictions. Theory also predicts that if clicking costs are zero, people should catch as many balls as possible and prizes should therefore not matter, which is what we observe. Thus, the ball-catching task also passes this second test.

The third and most demanding test is whether observed (average) behavior also follows point predictions. This is the case and thus the ball-catching task also passes this third test. We thus conclude from Study 1 that the ball-catching task is in principle suitable for theory testing purposes, if the researcher thinks that for his or her research question a design with tangible actions is desirable.

A complementary way of looking at the experiments reported in Study 1 is to see them as a test in its own right of piece-rate incentive theory. In its most simplified version, the first-order condition of optimal clicks under piece-rate incentives is expressed in Eq. (1). Our experiment provides an environment to put the comparative static predictions from (1) as well as clicking level predictions to a test. The experimental environment controls the production process (the ball dropping), the costs of clicking to catch balls, as well as the piece rates (the prizes) for each catch. Tests using field data, even those that have unusually detailed data such as Lazear (2000), typically do not have detailed information about effort costs that are necessary to predict effort levels. The ball-catching task can accommodate assumptions about effort costs (e.g. the cost consequences of ability differences) in the induced cost valuations given to subjects. The ability of the ball-catching task to control all aspects of the environment allows a complete behavioral characterization of all predictions of piece-rate theory, not just the comparative statics. Our results provide a comprehensive vindication of piece-rate theory.

Study 2 reported three experiments to showcase the implementation and versatility of the ball-catching task in three classic experimental paradigms that have been studied extensively in induced value experiments: team production, gift-exchange, and tournaments. In all three experiments the results are closely in line with findings from their induced value counterparts. Particularly noteworthy is that equilibrium predictions, derived from the production function of Study 1, are closely met in all cases where we could derive an equilibrium prediction (the piece-rate treatments of the team production experiment, and in the tournament). We also confirm the theoretical comparative static prediction that in the gift-exchange game a fixed matching should lead to stronger reciprocity than random matching. We see this as a strong encouragement for the suitability of the ball-catching task in potentially many more settings. The chosen experiments also demonstrate the versatility of the ball-catching task to manipulate the production technology and the cost function.

One central feature of the ball-catching task is its ability to control effort costs by inducing any effort cost function the experimenter deems appropriate. Recall that effort costs in economic models of labor supply denote any cost a worker might incur, physiological, psychological, or simply opportunity costs of foregone leisure. Existing real effort experiments model opportunity costs of effort by offering the subjects outside options, for example the opportunity to surf the Internet (Corgnet et al. 2015), to receive paid time-out for a few seconds (Mohnen et al. (2008)), to work on other productive individual tasks (van Dijk et al. 2001), or to leave the task earlier than the deadline (e.g., Abeler et al. (2011)). This method exploits the possibility of a trade-off between effort and off-the-job leisure and, indeed, there is experimental evidence that subjects make such a trade-off in response to different incentive schemes (see Corgnet et al. 2015; Eckartz 2014; Noussair and Stoop 2015). However, compared to the ball-catching task which in its most minimal version may take only one minute to complete, the “outside options” method usually requires a rather long duration for it to work well (sometimes up to 60 min as in Abeler et al. (2011)), thus preventing us from collecting repeated observations in the duration of a typical laboratory experiment. Moreover, while outside options imply some real effort costs, it is still unclear how subjects value them exactly without the help of structural estimation of the underlying effort cost function.^20^ The ability of the ball-catching task to induce any cost function, be it linear, or non-linear as in the gift-exchange experiment discussed above (Table 4), circumvents the problem of unknown valuations and retains the possibility of making point predictions on effort choices.

Studies 1 and 2 reported results of experiments conducted in the physical laboratory using z-Tree. Study 3 presented results from the online version of the ball-catching task, conducted on Amazon Mechanical Turk. The results strongly support the robustness of the ball-catching task with regard to all comparative statics predictions, including the crucial requirement of homogeneity of degree zero in *C* and *P*. This is encouraging and important support for the suitability of the ball-catching task.

However, the results from the online experiment also serve as an important caveat because they reveal that the environment where subjects take their decision might matter a great deal for the actual production function. In an online experiment, there are inevitably many differences compared to the physical laboratory: computer configurations (e.g., screen sizes and mice), speed of network connections, distractions in the working environment, etc. will vary strongly across online participants, but will typically be very similar for all subjects within a given physical laboratory. Physical labs might also differ, so the production function that can be used for deriving point predictions might also be lab specific. Hence, an important lesson is that for proper calibration of the production function pre-testing is necessary in whatever lab is used, physical or online.

## Conclusion

In this paper we introduced the ball-catching task, a task in which subjects can use mouse clicks to catch balls on screen, incurring material costs from each click. The task’s greatest advantage over related real effort tasks lies in its versatility to manipulate the production technology and in particular in its ability to control ‘effort’ costs. We presented three studies. Studies 1 and 3 showed that behavior in the ball-catching task in an individual decision making environment follows important comparative static predictions derived from incentive theory. Studies 1 and 2 suggest that the ball-catching task also has the potential to derive and test point predictions although Study 3 revealed that this most demanding feature of the ball-catching task requires careful calibration. Study 2 also showed that behavior elicited using the ball-catching task strongly resembles behavior in experiments using induced cost of effort designs. Together, the three studies demonstrate that the ball-catching task is a potentially powerful tool for (theory testing) experiments in “real effort” environments.

## Electronic supplementary material

Below is the link to the electronic supplementary material.

Supplementary material 1 (ZIP 37 kb)

Supplementary material 2 (MOV 3.06 mb)

Supplementary material 3 (PDF 541 kb)

Supplementary material 4 (ZIP 62 kb)

### Appendix

Here, we describe the working and functionality of the ball-catching task, both the z-Tree version and the online version, in more detail and also give suggestions about how to implement the task in experiments. The z-Tree code is available as online supplementary material. The online version is available from the authors upon request.

The z-Tree code of the ball-catching task allows experimentalists to manipulate the speed of falling balls and the time interval between falling balls directly in the global table in z-Tree. Changes to the layout of the task, such as the number of columns, height and width of the task box and the falling pattern, however, require more involved re-programming of the task. In the version used in this paper, the falling pattern is random. There are in fact four independent balls falling within a fixed time interval. Once a ball is caught or touches the bottom of the task box, it will reappear in a randomly selected column and fall again.

The z-Tree version has been tested using z-Tree 3.3.8 and later versions. The ball-falling and the tray-moving may become more sluggish with an increase in the number of z-Leafs simultaneously running the ball-catching task. In our experiments, we connected at most 16 z-Leafs to one z-Tree. A session with 32 subjects as in our Study 1 was accomplished by simultaneously opening two z-Trees in two separate master computers, each of which is connected with 16 z-Leafs. By affecting the level of sluggishness subjects may experience the number of connected z-Leafs may affect the production function. Other factors that may affect subjects’ performance include the size of the task displayed on the specific computer screen, pixel resolutions of computer monitors, mouse configurations, etc. It is, therefore, advisable to test the software thoroughly in the lab where the actual experiment will be run. This will help for calibration of the production function to allow for accurate point predictions.

The online version of the ball-catching task can be administered using a PHP/MySQL compatible server controlled by the experimentalist and a participant can enter the experiment using a JAVASCRIPT-enabled browser (modern browsers such as Firefox, Chrome, Safari and IE). As in the z-Tree version, the speed of falling balls and the time interval between falling balls can be easily changed in the program. The online version works differently from the z-Tree version in that there is a ball-generating mechanism that produces each ball with a fixed time interval from a randomly selected column. Therefore, unlike the z-Tree version, the distance between two balls falling near to each other is always the same. Because of the different engine behind the online version, participants typically do not experience any sluggishness in the ball falling and tray moving, although it may happen due to network connection issues or not fully JAVASCRIPT-compatible browsers.

The actual implementation of online experiments using this online version requires additional considerations compared to laboratory experiments. As discussed in the main text, performance of online participants, such as MTurkers, may be affected by technological and environmental considerations that are not observed by the experimenter. These include details of computer configurations (e.g., screen sizes and mice), conditions of network connectivity, as well as environmental distractions, etc..

## Footnotes

1 To our knowledge, one of the first experimental studies to use a real effort task for testing incentive theory is Dickinson (1999) in which subjects were asked to type paragraphs in a four-day period. Other early studies implementing real effort tasks within typical laboratory experiments include van Dijk et al. (2001), Gneezy and Rustichini (2000), Gneezy (2002), Konow (2000).

2 The z-Tree code as well as a demonstration video for the ball-catching task are downloadable from the journal website (supplementary materials to this article). Researchers are free to use this code (with appropriate attribution).

3 In our implementation subjects have a short amount of time in which to click and catch balls (one minute in Study 1). One could also implement the ball-catching task in a way that increases physical and cognitive costs and is perhaps more “effortful”, for example by increasing the time frame within which balls are caught.

4 Unless otherwise stated, in the version of the ball-catching task we use in this paper a maximum of 52 balls can be caught within 60 s.

5 The experiment was run in two sessions at the CeDEx lab at the University of Nottingham with subjects recruited using the online campus recruitment system ORSEE (Greiner 2015). Experimental instructions and data can be found in the online supplementary materials.

6 See an early review in Camerer and Hogarth (1999). Another possible reason for the “ceiling effect” is that subjects may also simply work on the paid task due to some experimenter demand effects (Zizzo 2010), particularly in the absence of salient outside options (see Augusto et al. (2015), Corgnet et al. (2015) and Eckartz (2014) for discussions).

7 We do not find any systematic change in average catches, average clicks or average earnings over the 30 periods. See the online supplementary materials for additional analysis of individual level data.

8 We also administered a post-experimental questionnaire where we asked subjects to rate the difficulty, enjoyableness and boredom of the task. On average, subjects reported that the task was very easy to do and they had neutral attitudes towards the enjoyableness and boredom of the task. Along with the quantitative data on clicks and catches, these responses are consistent with our interpretation that in our implementation of the ball-catching task psychological costs/benefits are not so important relative to pecuniary costs/benefits.

9 Fractional polynomials, which are an alternative to conventional polynomials, can afford more flexibility than conventional polynomials by allowing logarithms and non-integer powers in the models. The curve-fitting procedure used in the regression selects the best-fitting model with appropriate powers and/or logarithms. We also considered the possibility that the functional form might differ for *C* = 0 treatments, and so we fitted fractional polynomials excluding these data. We get the same specifications, and very similar coefficients.

10 The model specification of multiplicative terms with $Clicks_{i,r}^{0.5}$ implies that the conditional variation in catches is linear in clicks. We examined the relationship between clicks and squared residuals from a simple pooled regression of the model $Catches_{i,r} = \alpha_{0} + \alpha_{1} Clicks_{i,r}^{0.5} + \alpha_{2} Clicks_{i,r}^{2} + \pi_{i,r}$. We then regressed squared residuals on $Clicks_{i,r}$ as well as a nonlinear term (either $Clicks_{i,r}^{0.5}$ or $Clicks_{i,r}^{2}$). The coefficients on the nonlinear terms are not statistically significant, supporting our modelling specification of a linear relationship between conditional variation in catches and clicks.

11 To estimate model (2), note that dividing both sides by $Clicks_{i,r}^{0.5}$ transforms the model to a standard random effects model: $Catches_{i,r} /Clicks_{i,r}^{0.5} = \beta_{0} /Clicks_{i,r}^{0.5} + \beta_{1} + \beta_{2} Clicks_{i,r}^{3/2} + \delta_{r} + \omega_{i} + u_{i,r}$. Usual econometric techniques then follow.

12 See the online supplementary materials for details of the results: Estimates of model (2) for each session are given in Table B2 and the fitted production functions are shown in Fig. B2.

13 Note that we have assumed a continuous production function. This assumption is made mainly for expositional and analytical convenience. In reality, the production function is a discrete relationship between catches and clicks.

14 We also performed an out-of-sample test of predictions by comparing the actual number of clicks in an experimental session with the predictions derived from data from the other session. The results are essentially the same. See the online supplementary materials for details.

15 Four of the treatments were unrelated to this paper and are not reported. Instructions of all reported experiments are reproduced in the online supplementary materials.

16 This difference in variability of clicks between tournament and piece-rate incentives is smaller than that found by Bull et al. (1987) in their induced value experiment, in which the standard deviation of effort under tournament incentives was more than double that under piece-rate incentives. Quantitative comparisons between their study and ours, however, should be treated cautiously as there are numerous differences between studies (e.g. we use a piece-wise linear contest success function, whereas they use a rank-order tournament).

17 See the appendix for discussion of technical considerations associated with implementing the task.

18 See Horton et al. (2011) for a discussion of the usefulness of MTurk for experimental economists.

19 Analyses are available from authors upon request.

20 An alternative way to add realism to an experiment without sacrificing “control” over the cost function is to reduce the effort cost close enough to 0. For example, Abeler and Jäger (2015) used the slider task by Gill and Prowse (2012) but allowed the use of the keyboard, and derived lower bounds on the implied effort cost. The ball-catching task with the cost-per-click equal to 0 is in fact another example.
