# Supplementary material for: Combining “real effort” with induced effort costs: the ball-catching task
Source: Exp Econ. 2015 Sep 9;19(4):687–712. doi: 10.1007/s10683-015-9465-9 (PMC5153668; doi:10.1007/s10683-015-9465-9)
Supplement: Supplementary file 1 — Supplementary material 1 (ZIP 37 kb) [file 10683_2015_9465_MOESM1_ESM.zip › ztree-BCT/readme.rtf]

This folder contains all the z-tree codes for Studies 1 and 2.Study 1 —> piece-rateStudy 2 —> 1. team production                    2. gift exchange                    3. tournament
