# Supplementary material for: Combining “real effort” with induced effort costs: the ball-catching task
Source: Exp Econ. 2015 Sep 9;19(4):687–712. doi: 10.1007/s10683-015-9465-9 (PMC5153668; doi:10.1007/s10683-015-9465-9)
Supplement: Supplementary file 3 — Supplementary material 3 (PDF 541 kb) [file 10683_2015_9465_MOESM3_ESM.pdf]

Electronic Supplementary Material for

Combining "Real Effort" with Induced Effort Costs: The Ball-Catching Task

Simon Gächter, Lingbo Huang, Martin Sefton

University of Nottingham

Contents

A. Experiment Instructions
A1. Instructions Used in Study 1 p. 1
A2. Instructions Used in Study 2 p. 2
A3. Instructions Used in Study 3 p. 7
B. Additional Tables and Figures in Study 1 p. 9

A. Experiment Instructions

A1. Instructions Used in Study 1

Welcome to the experiment. You are about to participate in an experiment on decision making. Throughout the experiment you must not communicate with other participants. If you follow these instructions carefully, you could earn a considerable amount of money.

For participating in this experiment you will receive a £3 show-up fee. In addition you can earn money by completing a task. Your earnings from the task will depend only on your own performance.

You will be asked to work on a computerized ball-catching task for 36 periods. Each period lasts one minute. In each period, there will be a task box in the middle of the task screen like the one shown below:

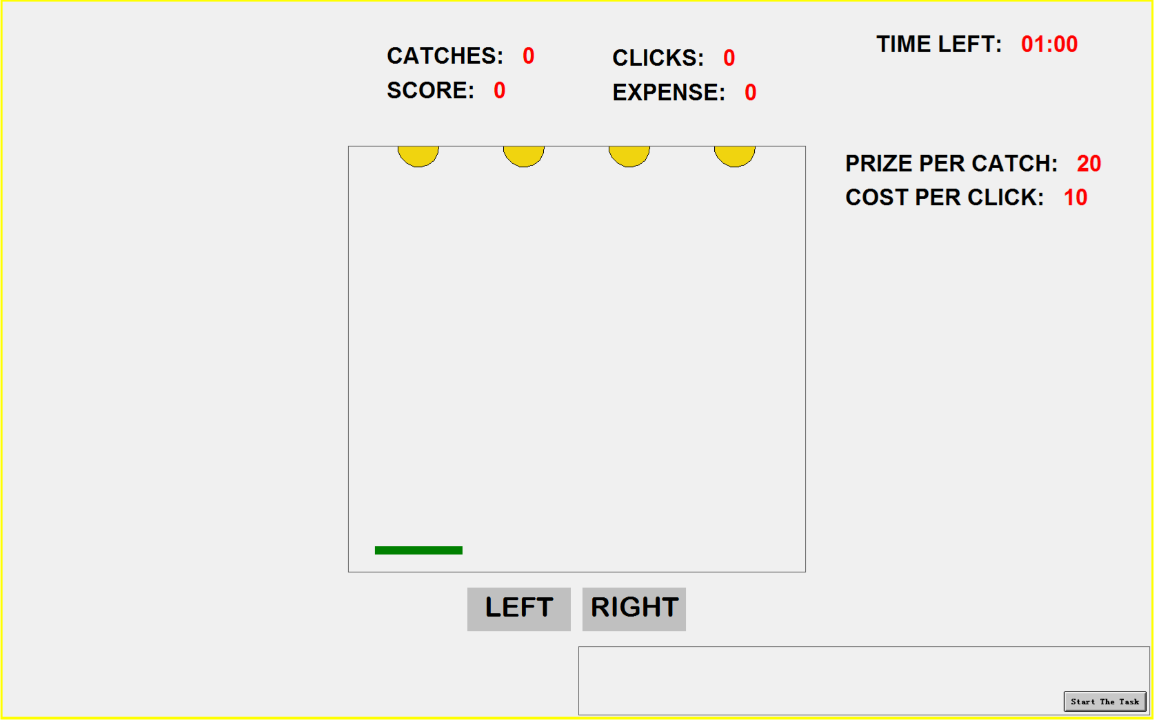

Once you click on the "Start the Task" button, the timer will start and balls will fall randomly from the top of the task box. You can move the tray at the bottom of the task box to catch the balls by using the mouse to click on the LEFT or RIGHT buttons. To catch a ball, your tray must be below the ball before it touches the bottom of the tray. When the ball touches the tray your catches increase by one.

You will receive a prize (in tokens) for each ball you catch and incur a cost (in tokens) for each mouse click you make. At the beginning of each period you will be informed of your prize for each ball caught, which will be either 10 or 20, and your cost for each click, which will be either 0, 5 or 10. In each period, the number of balls you caught so far (displayed as CATCHES), the number of clicks you made so far (CLICKS), your accumulated prizes so far (SCORE) and your accumulated costs so far (EXPENSE) are shown right above the task box. SCORE will be CATCH multiplied by your prize per catch for the period and EXPENSE will be CLICK multiplied by your cost per click for the period. At the end of the period your earnings in tokens for the period will be your SCORE **minus** your EXPENSE. Please note that catching more balls by moving the tray more often does not necessarily lead to higher earnings because both SCORE and EXPENSE matter for your earnings.

The first six periods will be practice periods which will not affect your earnings in the experiment in any way. At the end of the experiment, the tokens you earned from periods 7 to 36 will be converted to cash at the rate of 1200 tokens = 1 pound. You will be paid this amount in addition to your £3 show-up fee.

A2. Instructions Used in Study 2

Study 2 was run in five two-part sessions. Part One of sessions 1-5 implemented the Gift Exchange (Stranger), Gift Exchange (Partner), PR20, PR5 and Team Production treatments respectively. The Tournament treatment was conducted in Part Two of the fourth session. A pilot design that is not reported in this paper was conducted in the remaining parts.

General Information [All Treatments]

Welcome to the experiment. There will be two unrelated experiments for this session and there will be two separate instructions. The instructions for the second experiment will be distributed after the first experiment is ended. Throughout the session you must not communicate with other participants. If you follow these instructions carefully, you could earn a considerable amount of money. During the session your payment will be calculated in tokens.

At the end of each experiment tokens will be converted to cash at a rate of 1000 tokens = 1 pound. Your total payment for participating in this session will be the sum of your earnings in each experiment plus a £3 show-up fee. You will be paid this amount in cash at the end of the session.

If there is any question during the experiment, please raise your hand and someone will come to your desk to answer it.

[Team Production]

The first experiment has 10 periods. Before the first period, you will be randomly assigned to a group of four participants. You will be in this group for the entire experiment.

In each period, you and each of the other three participants in your group will be asked to work on a computerised ball-catching task. Your earnings in each period will depend on the number of balls caught by you and the rest of your group as well as some personal expenses as detailed below.

Your Task in a Period

Each period lasts one minute. In each period, there will be a task box in the middle of the task screen like the one shown below:

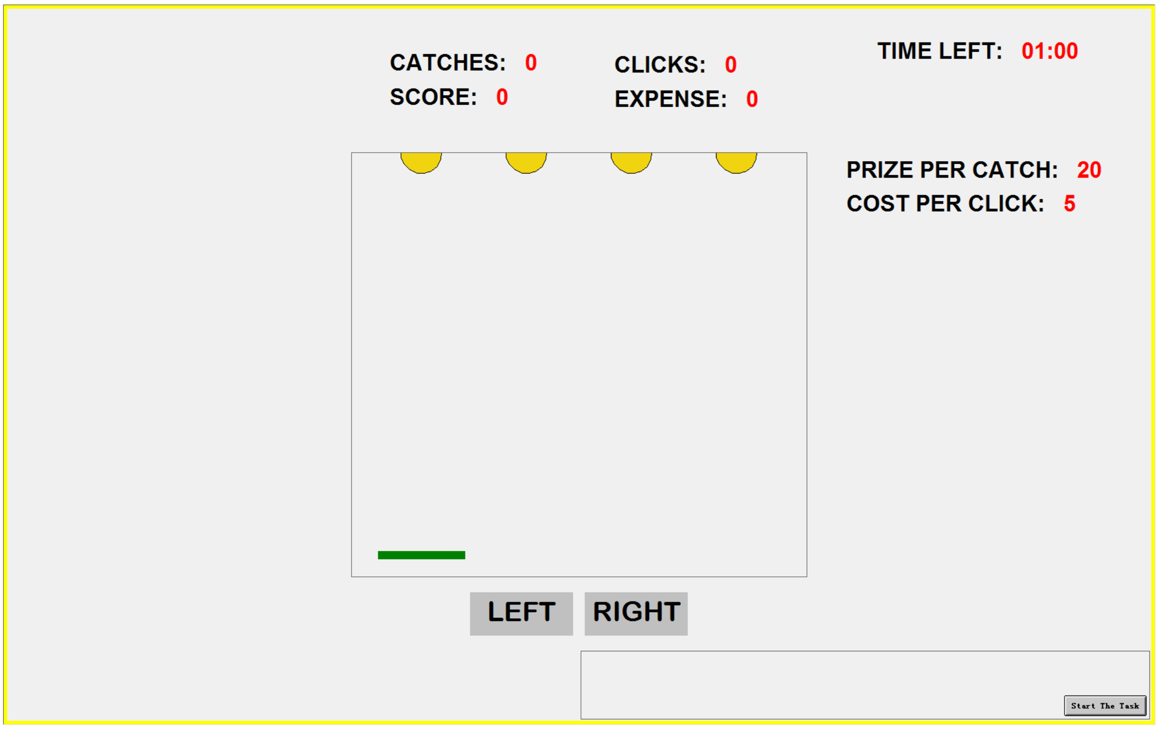

Once you click on the “Start the Task” button, the timer will start and balls will fall randomly from the top of the task box. You can move the tray at the bottom of the task box to catch the balls by using the mouse to click on the LEFT or RIGHT buttons. To catch a ball, your tray must be below the ball before it touches the tray. When the ball touches the tray your catches increase by one.

For each mouse click you make you will incur a cost of 5 tokens.

For each ball you catch, you and the rest of your group will in total receive a prize of 20 tokens. Similarly, for each ball each of your group members catches, you and the rest of your group will in total receive a prize of 20 tokens.

In each period, the number of balls you have caught so far (displayed as CATCHES) and the number of clicks you have made so far (CLICKS) will be shown right above the task box. Also shown above the task box will be SCORE, which is CATCHES multiplied by the prize per catch, and EXPENSE, which is CLICKS multiplied by the cost per click.

How Your Earnings In Each Period Are Determined

When you and the other members of your group have finished the task, the computer will calculate the TOTAL SCORE of your group by adding up the four individual SCOREs in your group. Your earnings in tokens will be one-fourth of your group TOTAL SCORE minus your EXPENSE:

Your Earnings = (your group’s TOTAL SCORE)/4 – EXPENSE.

Your SCORE and EXPENSE, your group’s TOTAL SCORE, and your earnings for the period will be displayed on the screen at the end of each period.

Quiz

1. Suppose you have caught 20 balls by making 10 clicks. The rest of your group have caught 100 balls.
   What will be your EXPENSE?
   Answer: _____ tokens
   What will be your SCORE?

Answer: _____ tokens
What will be your group's TOTAL SCORE?
Answer: _____ tokens
What will be your earnings in this period?
Answer: _____ tokens

2. Suppose you have caught 40 balls by making 40 clicks. The rest of your group have caught 20 balls.
   What will be your EXPENSE?
   Answer: _____ tokens
   What will be your SCORE?
   Answer: _____ tokens
   What will be your group's TOTAL SCORE?
   Answer: _____ tokens
   What will be your earnings in this period?
   Answer: _____ tokens

[Gift Exchange]

The first experiment has 10 paying periods. Before the first paying period, each participant will be randomly assigned to one of two groups: half will be "workers" and half "firms". You will remain either a worker or a firm throughout this experiment. In each paying period a firm will be randomly matched with a worker. Thus, [*Stranger treatment*: **you will be matched at random with another participant from period to period.** *Partner treatment*: **you will be matched with the same participant for the entire experiment**.] All firms and workers and the information on pairings will remain anonymous throughout the experiment.

Each paying period consists of two stages:

Stage 1: A firm will make a wage offer to the worker.
Stage 2: The worker will work on a task. The exact procedure is described below.

How do you calculate the firm's and worker's earnings in each paying period?

1. In each paying period, every firm and worker will receive 300 tokens.
2. Each firm may choose any integer number between 0 and 1000 as the wage in tokens that the firm offers to her paired worker.
3. After the firm has made a wage offer to her matched worker, the worker will be asked to work on a computerized ball-catching task. In each period, there will be a task box in the middle of the worker's task screen like the one shown below:

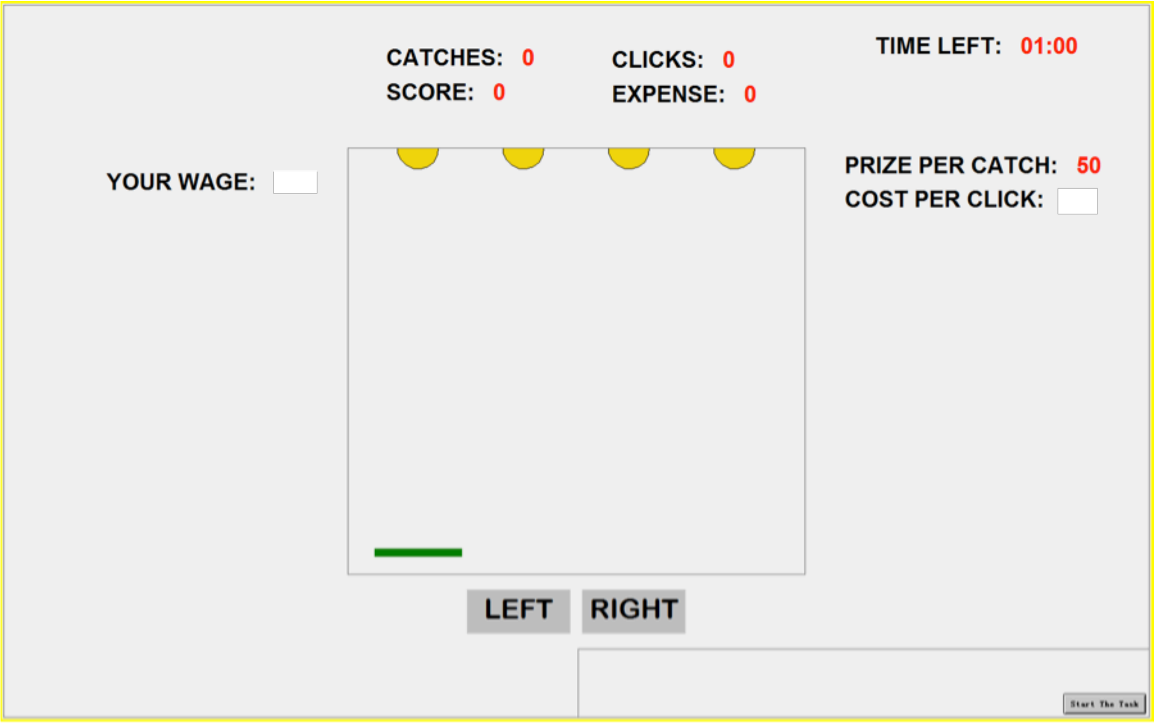

4. Each task lasts one minute. Once the worker clicks on the “Start the Task” button, the timer will start and balls will fall randomly from the top of the task box. The worker can move the tray at the bottom of the task box to catch the balls by using the mouse to click on the LEFT or RIGHT buttons. To catch a ball, her tray must be below the ball before it touches the tray. When the ball touches the tray the worker’s catches increase by one.
5. For each ball the worker catches, her matched firm will receive a prize of 50 tokens.
6. The worker will incur a specific cost for each mouse click she makes. The cost for each mouse click is shown below.

No.	1	2	3	4	5	6	7	8	9	10	11	12	13	14	15
Cost	5	5	6	6	6	7	7	7	7	8	8	8	8	8	9

No.	16	17	18	19	20	21	22	23	24	25	26	27	28	29	30+
Cost	9	9	9	9	10	10	10	10	10	11	11	11	11	11	12

For example, the column with No.6 means that the 6th click costs 6 tokens, the column with No.21 means that the 21st click costs 10 tokens, and finally the last column with No.30+ means that the 30th and any further click costs 12 tokens. Notice that if, for example, the worker makes a total of three clicks she will incur a total cost of 5 + 5 + 6 = 16 tokens.

7. The more clicks the worker makes, the more balls she may catch. Typically, if the worker decides to incur no cost by not moving the tray at all, she may still catch 4~6 balls. If the worker decides to catch every ball that she can, she may be able to catch 20 balls but she may need to click 20~30 times and incur a corresponding EXPENSE.
8. In each period, the number of balls the worker has caught so far (displayed as CATCHES), the number of clicks she has made so far (CLICKS) will be shown right above the worker’s task box. Also shown above the task box will be SCORE, which is CATCHES multiplied by the prize per catch, and EXPENSE, which is the total cost of CLICKS.

9. After the worker has completed the task, her earnings in tokens for the period will be determined by the following formula:

   Worker's Earnings = 300 + Wage – EXPENSE.

10. The firm's earnings in tokens for the period will be determined by the following formula:

   Firm's Earnings = 300 – Wage + SCORE.

Practice periods

Before starting the paying periods, there will be three practice periods in which every participant will play the role of the worker. These practice periods are meant to familiarise yourselves with the task, and to see how CLICKS are translated into CATCHES. The earnings in these practice periods will not affect your total payment for the experiment.

In each practice period, you will receive a wage, which is either 100, 500, or 900 tokens. You will then be asked to work on the ball-catching task as described above. Your earnings in each practice period will be calculated following the same rule above.

You will discover whether you will be a firm or a worker in paying periods after these practice periods.

Quiz

1. Suppose that a firm offers a worker a wage of 800 tokens. The worker catches 20 balls by incurring the EXPENSE of 300 tokens. What will be the firm's and worker's earnings respectively?
   Worker's earning = ______tokens
   Firm's earning = ______tokens

2. Suppose that a firm offers a worker a wage of 300 tokens. The worker catches 15 balls by incurring the EXPENSE of 160 tokens. What will be the firm's and worker's earnings respectively?
   Worker's earning = ______tokens
   Firm's earning = ______tokens

[Tournament]

The second experiment has 10 periods. Before the first period, you will be randomly paired with another participant. You will be in this pair for the entire experiment.

Your Task in This Experiment

In each period, you and your paired participant will complete the same ball-catching task as in the first experiment.

You will receive a score of 10 tokens for each ball you catch and incur a cost of 5 tokens for each mouse click you make. That means SCORE = 10×CATCHES and EXPENSE = 5×CLICKS

How Your Earnings In Each Period Are Determined

In each period, the person with higher SCORE in each pair will have a higher probability of being the winner. If you are the winner, you will earn 1200 tokens minus your EXPENSE for the period. If you are the loser, you will earn 200 tokens minus your EXPENSE for the period.

Your probability of winning will depend on the difference between your SCORE and that of your paired participant and some element of chance.

Specifically, say that the SCOREs of you and your paired participant are S1 and S2 respectively. Then your probability of winning the award is calculated as (S1 – S2 + 500)/1000 and the probability of winning of your paired participant is correspondingly calculated as (S2 – S1 + 500)/1000. That means, if the SCOREs are the same, both of you will have a 50% chance of being the winner. If the SCOREs are not the same, the chance of winning for the pair member with the higher SCORE increases by 1 percentage point for every increase of 10 in the difference between the SCOREs, while the chance of winning for the pair member with the lower SCORE correspondingly decreases by 1 percentage point.

Your SCORE, the SCORE of your paired participant, your EXPENSE, the EXPENSE of your paired participant, your probability of winning, whether you were the winner or the loser of the period and your earnings will be displayed on the screen at the end of each period.

A3. Instructions Used in Study 3

Welcome to the experiment. You are about to participate in an experiment on decision making. If you follow these instructions carefully, you could earn a considerable amount of money.

For participating in this experiment you will receive a $3.00 participation fee. In addition, you can earn money by completing a task.

You will be asked to work on a computerized ball-catching task for 36 periods. Each period lasts one minute. In each period, there will be a task box in the middle of your browser like the one shown below:

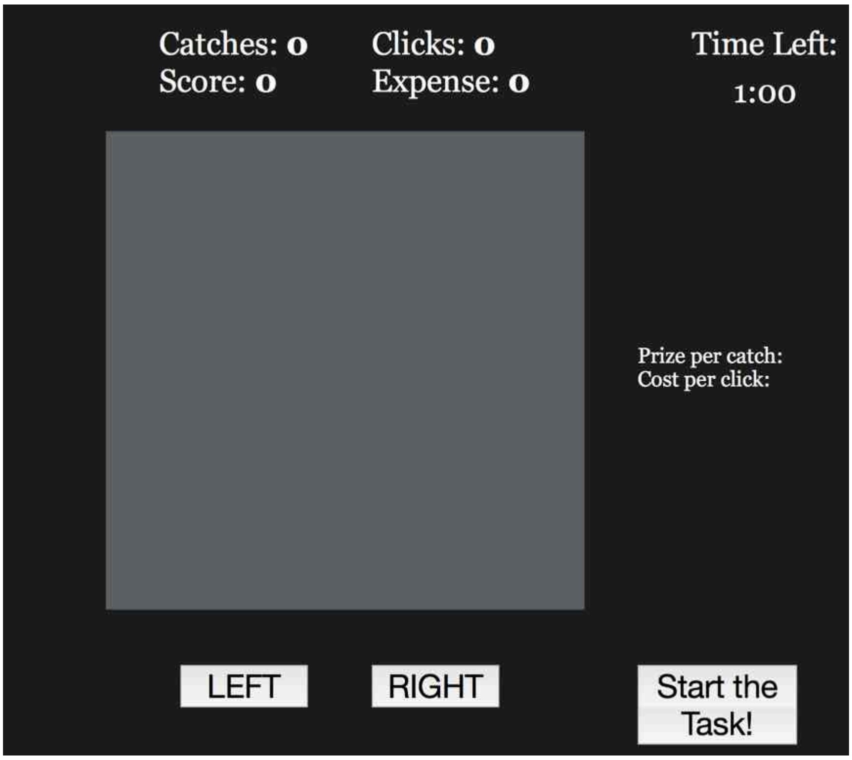

Once you click on the “Start the Task!” button, the timer will start and balls will fall randomly from the top of the task box. You can move the tray at the bottom of the task box to catch the balls by using the mouse to click on the LEFT or RIGHT buttons. To catch a ball, your tray must be below the ball before it touches the bottom of the tray. When the ball touches the tray your catches increase by one.

You will receive a prize (in tokens) for each ball you catch and incur a cost (in tokens) for each click you make. At the beginning of each period you will be informed of your prize for each ball caught, which will be either 10 or 20, and your cost for each click, which will be either 0, 5 or 10. In each period, the number of balls you caught so far (displayed as CATCHES), the number of clicks you made so far (CLICKS), your accumulated prizes so far (SCORE) and your accumulated costs so far (EXPENSE) are shown right above the task box. SCORE will be CATCH multiplied by your prize per catch for the period and EXPENSE will be CLICK multiplied by your cost per click for the period. At the end of the period your earnings in tokens for the period will be your SCORE minus your EXPENSE. Please note that catching more balls by moving the tray more often does not necessarily lead to higher earnings because both SCORE and EXPENSE matter for your earnings.

The first six periods will be practice periods, which will not affect your earnings in the experiment in any way. At the end of the experiment, the tokens you earned from the 30 paying periods will be converted to dollars at the rate of 5000 tokens = $1.00. You will be paid this amount in addition to your $3.00 participation fee.

B. Additional Tables and Figures in Study 1

Table B1 below reports the average number of clicks in each treatment for each individual. We also test whether each individual exhibited qualitatively consistent behavior, by which we mean that the subject's ranking of actual clicking is the same as the ranking of predicted clicks across all treatments. We predict the same level of clicking for treatments 2 and 6, and so we use an average of the actual clicking in these two treatments in our subject level test of consistency. Similarly, we predict the same level of clicking in treatments 1 and 4 and so the test uses the average clicks over these two treatments. Only 3 of the 64 subjects (less than 5%) behaved inconsistently: in all three cases clicking in treatment 5 is lower than in treatments 2 and 6.

Table B1: Average number of clicks in each treatment by subject

Subject	*1:(10,0)*	*2:(10,5)*	*3:(10,10)*	*4:(20,0)*	*5:(20,5)*	*6:(20,10)*	*Consistent behavior?*
101	59.8	27.4	12	64.6	45	27.6	√
102	62.6	18.4	12.2	63.6	30.2	20	√
103	51	14.8	9.4	65	22.8	14.2	√
104	21.6	6	3	20.2	5.4	4.4	√
105	62	15.8	3.6	60.6	18	16	√
106	56.8	18.8	9	58.6	34.8	19.2	√
107	57.2	20.8	11.2	57.8	29.6	26	√
108	47.4	13.4	11.6	54.6	25.8	10.8	√
109	62.6	18.8	9.6	65	26	14.2	√
110	60.2	20.8	7.2	61.4	39	23.8	√
111	61.4	5.8	5	63.8	9	7	√
112	48.6	6.4	2.8	49.2	8.4	6.8	√
113	64.6	25	16	69.8	48.2	29.6	√
114	49.2	17.4	14.6	59	24.2	16.8	√
115	64	18.8	9.2	64.4	30.2	21.4	√
116	63.8	29.8	11	66	52.6	29.8	√
201	56.4	36.2	13.2	53	39.2	34.6	√
202	38.2	9.4	4.6	38	11.6	7	√
203	61.8	19.2	11.4	58.4	27.6	17	√
204	64	24.4	6.8	55	37.8	23.2	√
205	65.4	21	10.6	58.4	37.2	16.6	√
206	64.6	24.6	11.4	61.6	45.6	25.8	√
207	67.2	31.4	14.8	60.8	53.2	32.2	√
208	53	19.6	6.8	53	27.8	17.4	√
209	51.2	28.6	5.4	49.2	43.6	22.2	√
210	72.8	10.6	8	65.2	16.8	10.6	√
211	62.4	20.8	8.8	63	40	22.4	√
212	63.2	23.4	10.2	68.4	53	28.6	√
213	63.4	20	12.2	58	31.4	19.2	√
214	65.8	30.4	18.8	64.2	24.2	25.4	×
215	59.2	24.8	13.6	53.4	36.6	25.2	√
216	58.4	7.6	3.8	59.6	31.8	12.2	√
301	61.2	3.4	3.2	60.8	5.6	13.4	×
302	63.2	16.4	9	70	21.8	13	√

303	46.8	17.2	11	50.8	32.2	11.6	√
304	38.2	5	1.4	30.6	5.6	1.4	√
305	52.4	14.2	7.2	60	23.6	13.2	√
306	67	14.6	7.4	63.2	36.2	13.6	√
307	56.6	16.8	5.6	64.4	29.6	17.2	√
308	44.6	29.4	8.6	48.6	27.8	28.6	×
309	52.8	7.4	4	56.6	37	9.4	√
310	44	17.2	9.8	50.2	29.8	21.8	√
311	52.6	15.8	7.4	66.4	29.6	11.8	√
312	69.4	13.8	6.2	62.4	26.4	9.8	√
313	60.8	17	7.4	71.6	40.4	23.6	√
314	56.6	23.6	10	64.6	56.8	24	√
315	58.4	21.8	8.2	63	23	16.2	√
316	63.2	15.8	15.2	59.8	23.4	18.6	√
401	59.6	13.2	13.2	64.4	15.6	16.8	√
402	65	20.8	8.6	66.2	42.2	23.2	√
403	41.2	16.2	10.4	42.8	15.4	13	√
404	57.2	24.4	5.2	60.2	38.4	27.6	√
405	51.6	16.8	8	54	25.8	15	√
406	62.4	25.8	11.6	69	58.4	29	√
407	67	8.2	2.8	69	10	6.8	√
408	67.6	17.2	7.2	62.6	28.6	13.8	√
409	62.4	16	4.8	58.6	42	14.6	√
410	47.4	11.4	8.6	35.6	13.6	11.6	√
411	54.4	13	6.4	49.4	20.4	13.6	√
412	66.4	34.8	0	62.6	52.2	41.4	√
413	71.8	13.8	13.4	67.6	23.2	16.8	√
414	62	27.6	9.8	64.2	45	30	√
415	48.4	16	11.8	55.6	22.8	15.2	√
416	67.8	37.2	11.8	70.4	66	17.8	√

Note: we consider the average clicks of treatment 2 and 6 and average clicks of treatment 1 and 4 when evaluating consistency at the individual level.

Fig. B1 Fitted production functions for sub-samples with different prizes using model (2)

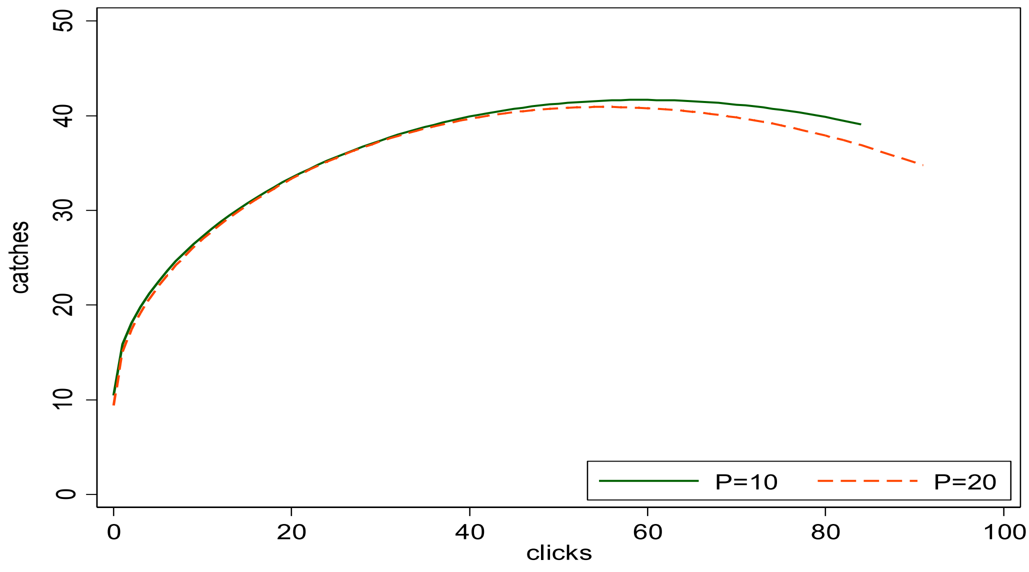

Fig. B2 Fitted production functions for sub-sample from different sessions using model (2)

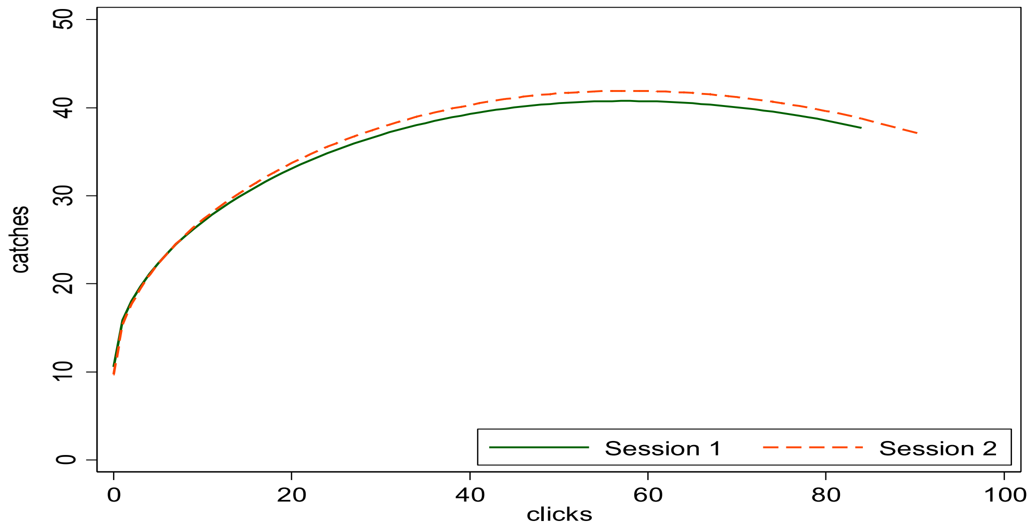

Table B2 Panel data regression estimates of model (2) for separate sessions

Dep. Var.: **Catches**	Coefficient estimates (std. err.)	
	(1) Session 1	(2) Session 2
$Intercept$	10.544***<br>(0.324)	9.730***<br>(0.326)
$Clicks^{0.5}$	5.315***<br>(0.189)	5.650***<br>(0.183)
$Clicks^{2}$	-0.003***<br>(0.000)	-0.003***<br>(0.000)
σ_{ω}	0.427***<br>(0.060)	0.287***<br>(0.046)
σ_{u}	0.785***<br>(0.018)	0.795***<br>(0.019)
N	959	946

Note: All period dummies are included and insignificant in both sessions. ***p < 0.01

Table B3 Comparisons between the predicted and actual number of clicks by session

**Treatment No.**	**1**	**2**	**3**	**4**	**5**	**6**
Prize per catch (P)	10	10	10	20	20	20
Cost per click (C)	0	5	10	0	5	10
Predicted clicks (S2)	57.6	20.1	7.3	57.6	35.1	20.1
Av. actual clicks (S1)	58.1	19.7	9.6***	58.2	31.5	19.6
(Std. Dev.)	(12.0)	(9.11)	(5.06)	(12.4)	(14.8)	(9.90)
Predicted clicks (S1)	57.4	18.7	6.5	57.4	33.9	18.7
Av. actual clicks (S2)	57.5	17.6	8.0***	59.2	30.3	17.3
(Std. Dev.)	(12.4)	(9.66)	(4.86)	(12.6)	(16.8)	(9.60)

Note: The full sample is equally separated into two halves by session: S1 and S2. We test the average actual clicks from S2 against the predicted clicks based on S1 and the average actual clicks from S1 against the predicted clicks based on S2. P-values are based on two-tailed one-sample t-tests against the predicted clicks using a subject's average clicks per treatment as the unit of observation. ***$p < 0.01$
