# Supplementary material for: Combining “real effort” with induced effort costs: the ball-catching task
Source: Exp Econ. 2015 Sep 9;19(4):687–712. doi: 10.1007/s10683-015-9465-9 (PMC5153668; doi:10.1007/s10683-015-9465-9)
Supplement: Supplementary file 4 — Supplementary material 4 (ZIP 62 kb) [file 10683_2015_9465_MOESM4_ESM.zip › Data/readme.rtf]

This folder contains all the data (in Stata format) and Stata do file that were used to produce all the figures and tables reported in all studies. The do file of Study 1 should be used for the data from Study 3.Study 1 —> piece-rateStudy 2 —> 1. team production                    2. gift exchange                    3. tournamentStudy 1 —> piece-rate online
